# Novel Benzene-Based Carbamates for AChE/BChE Inhibition: Synthesis and Ligand/Structure-Oriented SAR Study

**DOI:** 10.3390/ijms20071524

**Published:** 2019-03-27

**Authors:** Andrzej Bak, Violetta Kozik, Dariusz Kozakiewicz, Kamila Gajcy, Daniel Jan Strub, Aleksandra Swietlicka, Sarka Stepankova, Ales Imramovsky, Jaroslaw Polanski, Adam Smolinski, Josef Jampilek

**Affiliations:** 1 Institute of Chemistry, University of Silesia, Szkolna 9, 40 007 Katowice, Poland; violetta.kozik@us.edu.pl (V.K.); dkozakiewicz@us.edu.pl (D.K.); kamila.gajcy@us.edu.pl (K.G.); aswietlicka@us.edu.pl (A.S.); jaroslaw.polanski@us.edu.pl (J.P.); 2 Department of Bioorganic Chemistry, Faculty of Chemistry, Wrocław University of Science and Technology, Wyb. Wyspiańskiego 27, 50-370 Wrocław, Poland; daniel.strub@pwr.edu.pl; 3 Department of Biological and Biochemical Sciences, Faculty of Chemical Technology, University of Pardubice, Studentska 573, 532 10 Pardubice, Czech Republic; sarka.stepankova@upce.cz; 4 Institute of Organic Chemistry and Technology, Faculty of Chemical Technology, University of Pardubice, Studentska 573, 532 10 Pardubice, Czech Republic; ales.imramovsky@upce.cz; 5 Department of Energy Saving and Air Protection, Central Mining Institute, Plac Gwarkow 1, 40 166 Katowice, Poland; smolin@gig.katowice.pl; 6 Division of Biologically Active Complexes and Molecular Magnets, Regional Centre of Advanced Technologies and Materials, Faculty of Science, Palacky University, Slechtitelu 27, 78371 Olomouc, Czech Republic; josef.jampilek@gmail.com; 7 Institute of Neuroimmunology, Slovak Academy of Sciences, Dubravska cesta 9, 84510, Bratislava, Slovakia

**Keywords:** benzene-based carbamates, in vitro cholinesterase inhibition, CoMSA, IVE-PLS, molecular docking study

## Abstract

A series of new benzene-based derivatives was designed, synthesized and comprehensively characterized. All of the tested compounds were evaluated for their in vitro ability to potentially inhibit the acetyl- and butyrylcholinesterase enzymes. The selectivity index of individual molecules to cholinesterases was also determined. Generally, the inhibitory potency was stronger against butyryl- compared to acetylcholinesterase; however, some of the compounds showed a promising inhibition of both enzymes. In fact, two compounds (23, benzyl ethyl(1-oxo-1-phenylpropan-2-yl)carbamate and 28, benzyl (1-(3-chlorophenyl)-1-oxopropan-2-yl) (methyl)carbamate) had a very high selectivity index, while the second one (28) reached the lowest inhibitory concentration IC_50_ value, which corresponds quite well with galanthamine. Moreover, comparative receptor-independent and receptor-dependent structure–activity studies were conducted to explain the observed variations in inhibiting the potential of the investigated carbamate series. The principal objective of the ligand-based study was to comparatively analyze the molecular surface to gain insight into the electronic and/or steric factors that govern the ability to inhibit enzyme activities. The spatial distribution of potentially important steric and electrostatic factors was determined using the probability-guided pharmacophore mapping procedure, which is based on the iterative variable elimination method. Additionally, planar and spatial maps of the host–target interactions were created for all of the active compounds and compared with the drug molecules using the docking methodology.

## 1. Introduction

Several clinically implemented drug and pesticide molecules contain amide (–CONH–) and/or carbamate (–OCONH–) groups that can be variously substituted to form privileged structural fragments [1,2,3,4]. These molecular motifs are able to interact with a wide range of receptors/enzymes to induce a biological response [5,6,7]. As a result, a growing interest in amide- and carbamate-based compounds is being observed among medicinal chemists [8,9]. Based on the cholinergic hypothesis that assumes the increase in the acetylcholine (ACh) level, carbamate-like drugs can be regarded as one of the mainstays for the contemporary pharmacotherapy for Alzheimer’s disease (AD) by inhibiting cholinesterases (ChEs) [10]. AD is a progressive central nervous system (CNS) disorder that irreversibly degenerates the memory and cognitive abilities (dementia), which causes the everyday activities of older adults to be severely limited. Even though the etiology of AD has not yet been comprehensively revealed, a few factors are postulated as playing valid roles in the pathogenesis of this disease, including the aggregation and accumulation of amyloid-β peptide deposits in the brain, oxidative stress and a low level of ACh [11]. Basically, two types of the ChE enzymes that belong to the group of serine hydrolases occur in the human nervous system: acetylcholinesterase (AChE) and butyrylcholinesterase (BChE) [12]. In fact, both enzymes are able to catalyze ACh hydrolysis in the cholinergic synapses, but BChE can hydrolyze other esters as well; therefore, its concentration in the brain seems to be important for those suffering from AD. It is reported that AChE has a higher hydrolytic capacity, but its levels decrease as the changes become more pronounced during the course of the disease, while the BChE levels are supposedly increased or unchanged, which can somewhat compensate for the higher AChE activity [13]. Inhibiting both enzymes produces an increment of the ACh concentration in the cholinergic synapses that show a symptomatic efficacy in AD treatment; therefore, competitive cholinesterase inhibitors (ChEIs), e.g., galanthamine or rivastigmine as well as non-competitive ChEIs, e.g., tacrine or donepezil are clinically applied to alleviate the neuromuscular symptoms of AD [14]. In fact, novel BChE selective inhibitors such as anti-Alzheimer agents have recently been proposed, but the potential effectiveness that they offer is frequently accompanied by the occurrence of central and peripheral side effects [15,16,17,18]. In this context, further research for new ChEIs seems to be required to achieve success in the treatment of AD.

Potency modeling of prospective drug molecules seems to be useful as an initial screening test (rank order) and a decisive factor in avoiding “chasing” false positives and/or eradicating “bad actors” at the early stages of drug design or development [19,20]. The art of specifying molecules that have a potential therapeutic value at the “pre-synthesis” stage can be assisted by computer-aided molecular design (CAMD) techniques for the comprehensive mapping of the topology and/or topography of a compound into the property-based chemical space. A key question that confronts computational chemists is how to quantitatively transform a myriad of feature-based descriptors that have been a priori calculated for the compounds into an ADMET-related molecular potency. From among the more optimistic approaches, modeling the multidimensional quantitative structure–activity relationship (mD–QSAR) techniques seem to be pragmatic provided that a congeneric series of compounds is produced—the analogy is crucial in chemistry, especially in cases in which the investigated process is not fully understood or when the variety of observed guest–host interactions makes the optimization of the pharmacological response a cost/resources/knowledge challenging issue [21,22,23]. The “Holy Grail” of in silico modeling is to produce statistically robust models capable of making accurate quantitative predictions that include the binding affinity, metabolic fate and pharmacokinetic or toxicity parameters (ADMET) on the route “towards the prediction paradise” [24]. Hence, huge attempts have been undertaken to implement “direct” receptor-dependent (RD) and “indirect” receptor-independent (RI) in silico protocols, respectively. The working paradigm of fragment-based design, or fragonomics, assumes that well-placed fragments account for the crucial ligand–receptor interactions, while the rest of the molecule primarily serves as a scaffold that holds the “active” fragments together [25]. With regard to the structure of a ligand as a “negative” image of an active target site (pharmacophore), the question about a reliable quantitative measure of its “intrinsic” activity and its correspondence to robust empirical receptor–ligand interaction data naturally appears. In the target-guided QSAR procedures. the complementary (bio)effector-binding mode is retrieved based on the intrinsic dependence of the atomic coordinates of both the receptor and ligand in the binding/active site while the target spatial arrangement of the atoms is also available [26]. The adopted spatial distribution of the ligand property space is mediated by the corresponding mapping of the target steric, electronic or lipophilic patterns. In fact, drugs are generally engineered to have a tightly fitting interface with the receptor/enzyme bonding/active site; however, the “goodness” of a favorable guest–host binding assessment is still questionable. In other words, the Achilles’ heel of docking procedures is the deficiency of “truly” selective scoring functions to rank the receptor–ligand complexes according to their actual binding affinities [27,28]. To overcome these constraints, it is advisable to use both ligand- and structure-based approaches on the path from the relationships to the models.

The current paper can be regarded as a follow-up of the recently reported carbamates as prospective ChEIs [29,30,31]; therefore, the synthesis and biological assessment of a new series of benzene and naphthalene derivatives is provided. Moreover, the electronic/steric/lipophilic determinants, which are potentially valid for characterizing the structure-inhibitory potency for a set of the carbamate analogs, are discussed using RI and RD in silico procedures: comparative molecular surface analysis (CoMSA) and molecular docking [32]. Additionally, a systematic space inspection was performed to investigate the statistical estimators using the stochastic model validation (SMV) method to derive the “average” pharmacophore pattern in the probability-guided pharmacophore approach [33]. The molecular pharmacokinetic profiles were comprehensively screened using several descriptors that revealed the physicochemical and structural features, which might be essential to map the inhibition potency of carbamates. 

## 2. Results and Discussion

### 2.1. Design and Synthesis

**Scheme 1:**
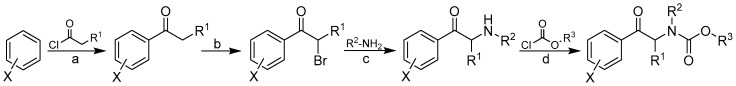
Synthesis of benzene derivatives **1**–**34**. Reagents and conditions: (**a**) AlCl_3_, CH_2_Cl_2_; (**b**) Br_2_, CH_2_Cl_2_; (**c**) HCl; and (**d**) CH_2_Cl_2_.

**Scheme 2:**
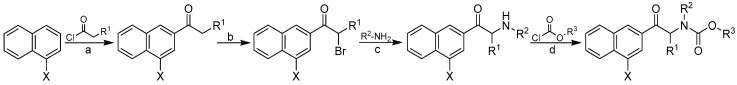
Synthesis of the naphthalene derivatives **35**–**41**. Reagents and conditions: (**a**) AlCl_3_, CH_2_Cl_2_; (**b**) Br_2_, CH_2_Cl_2_; (**c**) HCl; and (**d**) CH_2_Cl_2_.

The concept of the synthesis of the designed compounds was based on a modification of the structure of the selected α-aminoketones. Cathinone is biologically active natural product and its derivatives have broad pharmacological properties. All carbamates were formed in a four-step synthesis that begins with the basic reagents (see Scheme 1 and Scheme 2). 

In the first step, the corresponding aromatic ketone was obtained in the Friedel–Crafts reaction. Next, the bromination of the obtained aromatic ketone with elemental bromine led to a 2-bromo derivative, which was subjected to the ammonolysis of the obtained halide without secretion. In the final step, the amine in a free form reacted with the appropriate chloroformate to produce desired carbamate. The final products were purified using column chromatography with a mixture of ethyl acetate and hexane, respectively. The structures of the obtained compounds are presented in Table 1. All of the derivatives were analyzed in order to confirm their chemical structures and were also tested for their biological activity.

### 2.2. In Vitro Assessment of the AChE- and BChE-Inhibitory Profiles

The AChE- and BChE-inhibiting activity for all of the tested carbamates was evaluated and compared with the internal standards rivastigmine (RIV, Exelon^®^) and galantamine (GLT, Reminyl^®^). These standards were selected because of their different structures since rivastigmine is a classical acylating pseudo-reversible carbamate ChEI that inhibits both AChE and BChE, whereas galanthamine is a non-acylating competitive reversible ChEI as well as an allosteric ligand at the nicotinic ACh receptors. The choice of these reference drugs, which have different mechanisms of action, can provide relevant results. The findings are summarized in Table 1 and expressed as the 50% inhibitory concentration (IC_50_ [μM]) or the concentration of the inhibitor that was required for the 50% inhibition of the mentioned enzymes. 

All of the considered derivatives exhibited a very good to moderate inhibitory activity. The IC_50_ values for AChE ranged from 32.01 ± 0.54 to 329.68 ± 42.06 µM and for BChE from 5.51 ± 0.20 to 440.56 ± 7.54 µM. It was observed that some of them had an even better activity than a known drug—rivastigmine. Surprisingly, more of the compounds exhibited a better inhibitory potency towards BChE than to AChE. Some of the compounds proved to be highly selective for BChE with respect to AChE (**11**, **13**, **14**, **16**, **23**–**28**, **31**, and **33**). Specifically, two compounds, **23** and **28**, had a very high selectivity index (SI = 13.93 and 15.31). In fact, compound **28** had the lowest IC_50_ value and had an approximately seven-fold higher inhibitory activity against BChE than RIV (IC_50_ = 5.51 vs. 38.40 µM), which corresponds quite well with GLT (IC_50_ = 2.77 µM). Similarly, a five-fold higher inhibitor activity against BChE relative to RIV was also observed for compound **31** (IC_50_ = 7.02 µM) and was more than three-fold higher for compounds **23** and **32** as well. It should be emphasized that compound **32** exhibited a promising inhibitory activity against AChE with an IC_50_ value of 32.01 µM—approximately two times better compared to RIV (IC_50_ = 56.1 µM). Among the naphthalene derivatives, phenyl carbamates **36**–**38** revealed a three-fold higher inhibitor activity towards BChE relative to RIV, whereas only one benzyl carbamate, **39**, exhibited a two-fold higher anti-BChE activity to RIV. In fact, compounds **36** and **39** might serve as selective inhibition agents towards BChE with respect to AChE (SI = 7.83 vs. 5.55). Moreover, compounds **37** and **38** also exhibited a high activity against AChE. Not surprisingly, the substitution of the aryl ring had a direct impact on the variations in the potency of the investigated compounds. It was observed that the methyl-substituted carbamates generally exhibited a lower inhibitory ability compared to their phenyl or benzyl counterparts. Interestingly, compounds **17** and **29**, which have the methoxy group in the same position were weaker inhibitors—the presence of a hydrophilic electron-donating -OCH_3_ substituent of the phenyl ring at the *para*-position decreased the potency of the compounds, especially against the BChE enzyme (see Table 1). On the other hand, the presence of methyl group(s) in the meta/para-position(s) of the phenyl ring (compounds **15**, **18**, **21**, **27**, **30**, and **33**) resulted in an improvement in the IC_50_ value for the BChE enzyme, thus suggesting the significance of the hydrophobic interactions with the enzyme. The placement of an electron-withdrawing chlorine substituent in the meta-position of the phenyl ring appeared to be strongly preferable, especially for the BChE inhibition activity (compounds **16** or **28**).

### 2.3. In Silico Evaluation of AChE- and BChE-Inhibitory Profile

**Figure 1:**
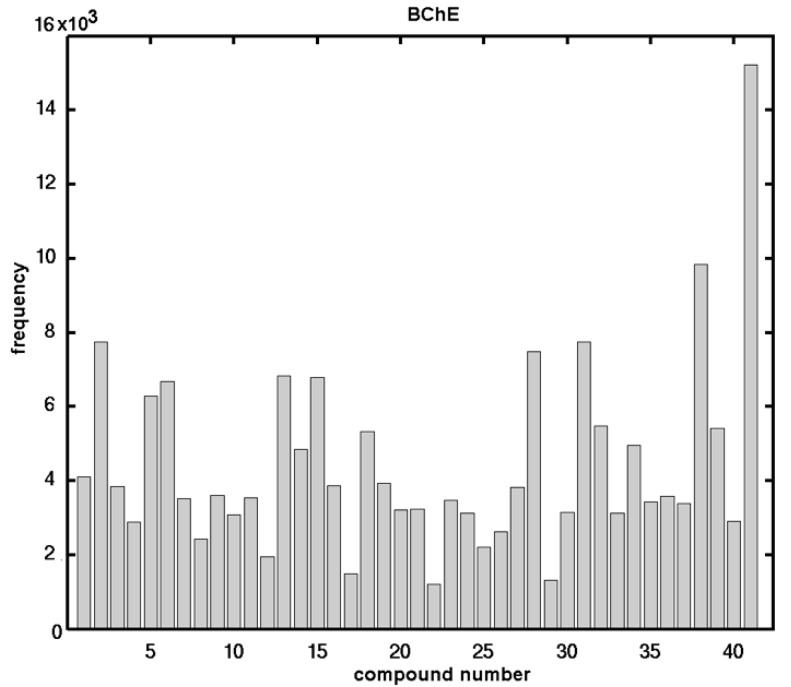
Histogram indicating number of individual compounds that accured in test set within the regions of $q_{cv}^{2}$ > 0.7 and $q_{test}^{2}>$ 0.5 for the training set against $q_{test}^{2}$ L–7–O CV for modeling the BChE potency of the carbamate derivatives (**1**–**41**) using CoMSA approach.

The principal objective of the ligand-based study was to comparatively analyze the molecular surface (CoMSA) to gain insight into the electronic and/or steric factors that potentially determine the inhibitory AChE/BChE activities of the investigated compounds. The findings for the surface descriptors were compared with their force field counterparts (CoMFA), when modeling thinhibiting potency for the multiple training/test subsets. First, the $q_{cv}^{2}$ performance of the AChE and BChE profiles for the entire benzene and naphthalene carbamate dataset **1**–**41** was investigated as the training set using the CoMFA and CoMSA approach. In this case, both methods performed comparably for the AChE/BChE activity (expressed in a logarithmic scale as pAChE and pBChE); however, in general, the modeling of pBChE data produced superior outcomes of the statistical metrics (CoMFA $q_{cv}^{2}$ = 0.61 vs. CoMSA $q_{cv}^{2}$ = 0.61), irrespective of the map size (20 × 20 to 50 × 50) or the probe atom that was used (CH_3_^+^ or H^+^). It was clear that relying exclusively on data fitting with cross-validated leave-one-out (CV-LOO) procedure is not satisfactory, and therefore the external validation by splitting the molecules into training/test subsets was conducted to assess the predictive power of a model using the SDEP, MAE and $q_{test}^{2}$ statistics. The naphthalene-based carbamates were excluded from the training set in order to form the test set. We observed an improvement in the $q_{cv}^{2}$ performance (CoMFA $q_{cv}^{2}$ = 0.72 vs. CoMSA $q_{cv}^{2}$ = 0.76); however, the poor predictive abilities of the models ($q_{test}^{2}$ < 0.5) confirmed the dichotomic nature of the $q_{cv}^{2}$/$q_{test}^{2}$ parameters for which a high value of $q_{cv}^{2}$ did not automatically imply a high model predictability [34]. From a philosophical point of view, it is not possible to determine an “absolute” measure of predictivity, as it significantly depends on the choice of datasets and the statistical approach that is used, but the great advantage of the QSAR paradigm lies not in the extrapolation, as was stated by Hansch [35]. In other words, the separation of objects into training/test subgroups is not a trivial issue, and therefore, an additional evaluation, namely the Stochastic Model Validation (SMV), was performed as a kind of “perturbation” procedure to examine the data structure [36]. Hence, the fluctuations of the statistical estimators during the CoMSA BChE modeling were examined since the original dataset of 41 molecules was repeatedly sampled into 30/11 training/test subseries (ratio 3:1). Unfortunately, it was not technically feasible to scrutinize the entire pool of systematically generated training/test populations ($C_{41}^{11\;}$≈ 3 × 10^9^), and, therefore, the overall number of samplings was restricted to a relatively small fraction of approximately 3 × 10^6^ systematically generated populations (1 out of 1000). Not surprisingly, the generated $q_{cv}^{2}$ vs. $q_{test}^{2}$ fluctuation pattern revealed the areas with a greater modeling ability that were depicted for the BChE potency ($q_{cv}^{2}$ ≥ 0.75) and were accompanied by areas that had a higher ($q_{test}^{2}$ > 0.5) or lower ($q_{test}^{2}$ < 0.5) predictive power. Obviously, the preferential selection of objects into the training set resulted in a decrease in the predictive performance of the test set. Figure 1 illustrates the frequency distribution of the compounds in the test subgroup when sampling the models that had the best statistical parameters ($q_{cv}^{2}$ ≥ 0.7 and $q_{test}^{2}$ > 0.5). A relatively smooth compound frequency distribution within the test set population was disrupted by the larger numbers of compounds **2**, **5**, **6**, **13**, **15**, **28**, **31**, **38**, and **41**, respectively.

In general, the specified molecules covered the entire structural space as well as the BChE activity range (5.51–440.65 µM) of the investigated compounds evenly, which partially explains the good ability and predictability of the model.

Additionally, the similarity was analyzed using the PCA procedure on the pool of descriptors that was derived from Dragon 6.0 software, in which the final dataset of 2777 variables was arranged in matrix X_41×2777_ with the rows representing molecules (objects) and the columns preserving the numerical values of the variables (parameters) [37,38]. The experiment was carried out for the centered and standardized data to illustrate any meaningful variations in the modeling performance of the examined set of carbamate derivatives with respect to their structure and inhibitory profiles. The PCA model with the first four PCs described 80.66% of the total data variance, whereas the first two PCs accounted for 67.37%. 

**Figure 2:**
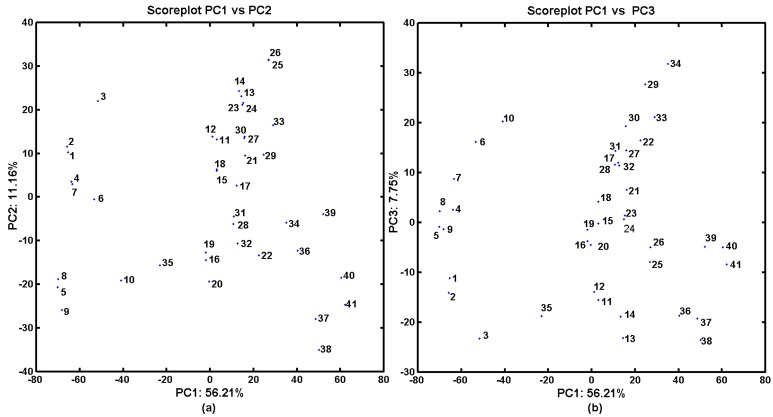
Projection of carbamates **1**–**41** on a plane defined by two principal components for the Dragon descriptors: the first vs. second (**a**); and the first vs. third (**b**).

Figure 2 presents the respective score plots of PC1 vs. PC2 (Figure 2a) and of PC1 vs. PC3 (Figure 2b), which indicated that the methyl-based carbamate derivatives (**1**–**10** and **35**) can be classified into one structurally related group along the first principal component (PC1 < −20). Moreover, the naphthalene-based derivatives were generally grouped together (PC1 > 40), which indicated major structural variations from all of the remaining ones. 

**Figure 3:**
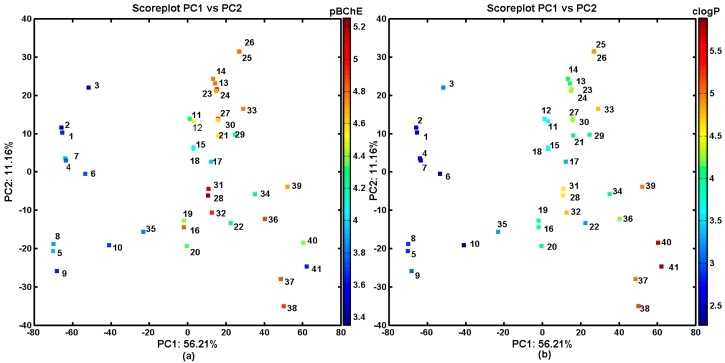
Projection of carbamates **1**–**41** on the plane defined by the first vs. second principal components with: BChE activity in the logarithmic scale (**a**); and the calculated lipophilicity profile (**b**). Colors indicate the numerical values of the pBChE activity and clogP.

A detailed inspection of the experimental BChE inhibitory profile and compound lipophilicity, which were color-coded according to the values of logP (calculated in Sybyl-X) for the objects that were projected on the plane specified by the two first components, revealed some correlation (R = 0.67). This was especially visible for the methyl-based carbamates (**1**–**10** and **35**), for which the was the lower activity (pBChE < 4) the lower was the lipophilicity (clogP < 3). On the other hand, some of the more active naphthalene derivatives were accompanied by fairly high values of the calculated lipophilicity (clogP > 5) as observed in Figure 3. 

**Figure 4:**
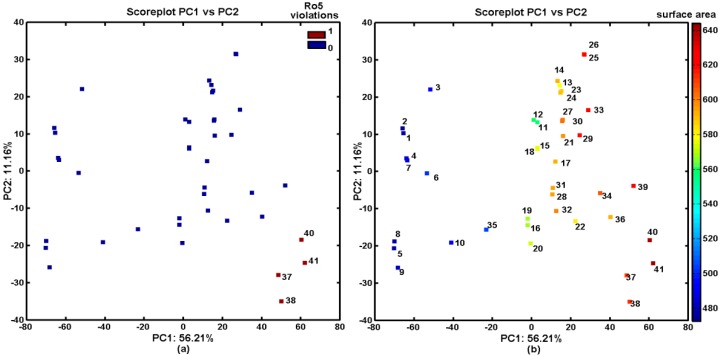
Projection of carbamates **1**–**41** on the plane defined by the first vs. second principal components with: Ro5 rule violations (**a**); and the calculated surface area (**b**). Colors indicate the number of Ro5 violations and the numerical values of surface area.

One can conclude that the lipophilic profile for compounds should be directly related with their chemical structure, and, therefore, structurally similar molecules (chemotypes) might have similar properties. This tendency can be confirmed for halogenated naphthalene derivatives (**37**, **38**, **40**, and **41**), which violated the Lipinski’s Rule of Five (Ro5), as can be observed in Figure 4a. Obviously, the above chlorine- and bromine-based molecules were characterized by the greatest values of the surface area descriptors, which were obtained by the Sybyl-X software, as visualized in Figure 4b. 

### 2.4. Probability-Guided Pharmacophore Mapping 

**Figure 5:**
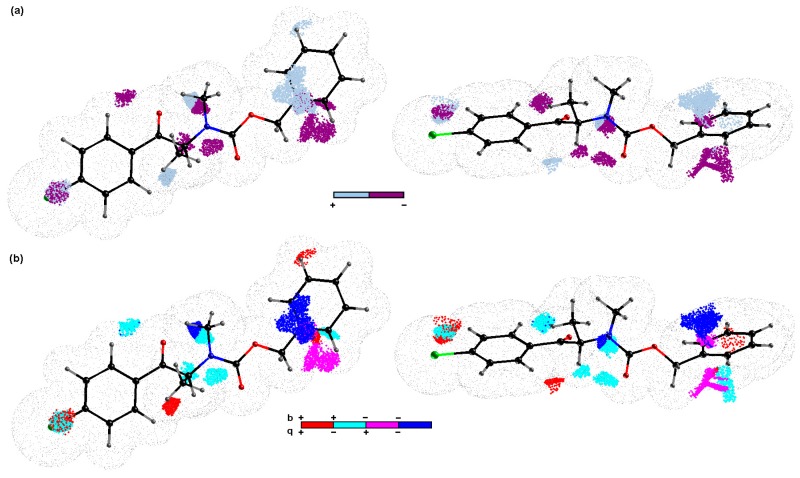
The spatial sectors with the greatest contribution into the BChE inhibitory potency specified as CoMSA IVE-PLS for the selected 30/11 training/test set samplings. Colors indicate their effect (**a**) and the four possible combination of the mean charge q and correlation coefficient b values (**b**). Reference compound **32** is plotted in two different orientations.

It is characteristic that a vast set of highly inter-correlated topologic/topographic descriptors is generated for mD-QSAR studies, and, therefore, the uninformative variable elimination procedure can be regarded as a pre-processing stage to prune the input data ensemble. As a result, the iterative variable elimination procedure (IVE-PLS) was used as a filter to specify the descriptors with the highest individual weightings towards the inhibitory activity [39]. Thus, 10% of the 30/11 training/test samples was randomly selected from regions that had a fairly high model ability ($q_{cv}^{2}$ > 0.70) and predictability ($q_{test}^{2}$ > 0.5) to produce “consensus-based” pharmacophore maps. Taking the number of molecules into account, the maximum number of PLS components to generate the model was truncated to seven. The variables with the highest stability *abs(mean(b)/std(b))* for each of the randomly selected models were specified using the IVE-PLS algorithm. In general, the predictive power of a model that was monitored by $q_{test}^{2}$ was stable for a considerable range of the variables that were eliminated, whereas there was a slight improvement of the $q_{cv}^{2}$ performance when the columns with the lowest values of stability were extracted. The backward column elimination was iteratively looped until the optimal number of variables to be included within the model was accomplished—the moment that the $q_{cv}^{2}$ deterioration determined the number of relevant columns. The cumulative sum of the common columns for all of the investigated 1661 BChE models was calculated and normalized to a range of <0,1>. The set of columns with a value above the pre-selected cut-off of 0.4 was selected; however, the spatial pattern, as depicted in Figure 5, was produced by filtering a further 80% of the CoMSA descriptors that had a relatively small statistical significance for the BChE inhibitory activity. To simplify the visual inspection of the key pharmacophore patterns, colors were used to indicate the impact of the descriptors on the inhibitory potency to show the areas with a positive and/or negative activity contribution (Figure 5a) as well as the four possible combinations of the mean regression coefficients and charge values (Figure 5b), respectively. 

The spatial areas with a potentially detrimental impact on the BChE inhibitory potency, primarily due to steric or electrostatic factors, are indicated by the dark spheres in Figure 5a, whereas the bright polyhedrals depict the 3D pattern that was predicted to be positioned by an atom or substituent to reinforce the molecular inhibitory profile. It appeared that the elongation of the side chains R^1^ and R^2^ bonded to the peptide-bond-like motif in the scaffold contributed favorably to the BChE inhibition activity of the analyzed carbamates, as was suggested by the bright areas close to the nitrogen attached directly to the carbonyl group. This confirms the tendency that was observed among the carbamate homologs in which an additional methylene group improved the BChE inhibition affinity slightly (see Table 1). The obtained findings demonstrate the significance of the side chain R^3^, where the mixed (positive/negative) steric contribution to the inhibitory potency was observed, as shown in Figure 5a. The increase in the bulkiness in this area appears to be a favorable structural variation that partially elucidates the increase in the BChE potency for phenyl and benzyl carbamates compared to the methyl counterparts, as presented in Table 1. The presence of negatively charged motifs (delocalized π electrons) probably contributed favorably (negative regression coefficients) to the inhibition profile of the analyzed series, as depicted in Figure 5b. Basically, the spatially disallowed areas that were attributed by the negative mean charge values of the IVE-PLS CoMSA models were specified for the *para* position of the phenyl ring (X substituent). In other words, the indicated area might have been occupied by negatively charged atoms/groups, but an increase in the volume/surface may be detrimental to the inhibition potential, which corresponds quite well with the depletion of the BChE inhibitory abilities that were observed for the bromine- vs. the chlorine-based carbamates (see Table 1).

The visualization of the pharmacophore maps, which were generated using the consensus 3D-QSAR methodology, provided valuable knowledge about the interaction mode that was required to enhance the inhibitory potential of the analyzed carbamates as prospective ChEIs.

### 2.5. Molecular Docking 

Site-directed computer-assisted docking algorithms optimally position a (bio)effector molecule in a binding/active site, which is based on the intrinsic relationship of the target–host atomic coordinates, while the spatial geometry, or at least the homology models of macromolecule, are accessible [40]. In fact, target-guided docking algorithms mainly employ descriptor-matching approaches in which the ligand property space is adapted to the corresponding map of the target electronic, steric and lipophilic features. How to in silico estimate the “natural” forces that combine the building blocks together in structure-based drug-design (SBDD) is still unclear, since the method does not always produce a reliable quantitative correlation between the experimental and predicted activity assays [41]. In fact, the computational procedures that attempt to reconstruct a ligand–receptor complex using the molecular docking approaches involve an efficient search procedure and effective scoring function, respectively [42]. Rapidly covering the relevant conformational space at the search stage as well as the effective discrimination between native or non-native docked conformation at the scoring stage are basically the two critical elements of the docking procedure; however, the SBDD methodology is typically used for hit virtual screening (flash docking) and lead to optimization [43,44]. 

The geometries of human acetyl (AChE) and butyrylcholinesterase (BChE) enzymes that are co-crystallized with pharmacologically used drugs (e.g., galanthamine or rivastigmine) are still under intense scrutiny, which enabled us to compare the findings of the ligand- and structure-based protocols. On the other hand, a comprehensive examination of the guest–target interactions using molecular dynamic simulations (MDs) was beyond the scope of this study. 

**Figure 6:**
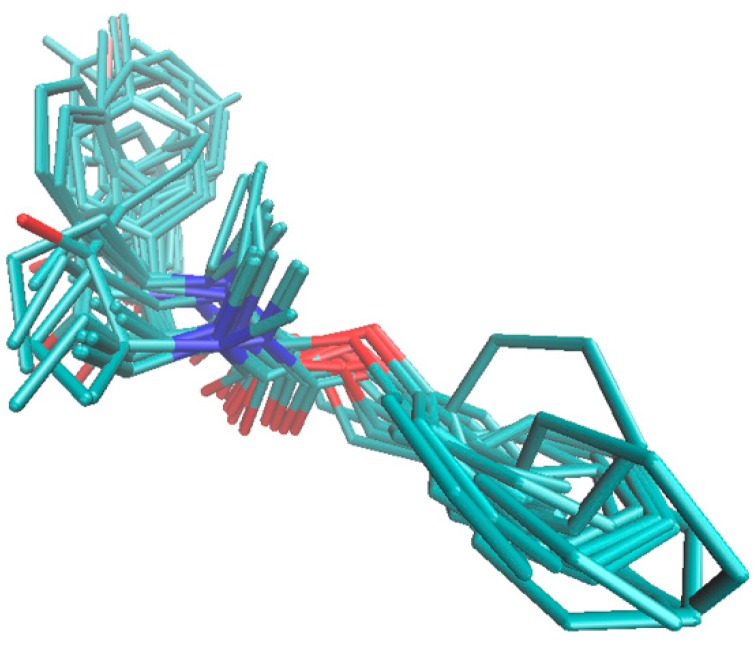
Comparison of the poses for the most active BChE inhibitors in the enzyme active site AC2. As an additional experiment galanthamine (GLT), which is a very active AChE/BChE inhibitor (IC_50_ < 3 µM) was docked in the enzyme active site AC2. Although the structural variations between GLT and RIV and the most active molecules are easily noticeable, similar spatial patterns of the host–BChE complex within the catalytic core was observed for the negatively charged atoms (nitrogen and oxygen), as presented in Figure 7.

The crystallographic data of butyrylcholinesterase with a catalytic core that is specified at a higher resolution of 2.6 Å in the liganded state (holo) with the rivastigmine (RIV) analog ([3-[(1~{R})-1-(dimethylamino)ethyl]-4-oxidanyl-phenyl]~{N}-ethyl-~{N}-methyl-carbamate) were retrieved from the Protein Data Bank repository (PDB entry: 6eul). The drug analog was subsequently (re)docked in the active site AC2 of the enzyme chain A, which is composed of six amino acid residues (Asn68, Ile69, Asp70, Trp82, Thr120, and Pro285) and a 1,2-ethanediol molecule (EDO605), using the AutoDock Vina program [45]. Moreover, the population of the BChE inhibitors (IC_50_ < 30 µM) that are comparatively active to rivastigmine was investigated to compare the interacting mode of the carbamate derivatives with the drug–enzyme spatial interaction pattern. In fact, the specific conformations and orientations (poses) of the most active compounds (**11**, **13**, **14**, **16**, **23**–**28**, **30**–**33**, and **36**–**39**), as illustrated in Figure 6, confirmed our intuitive selection of the trial alignment in the ligand-based study. 

**Figure 8:**
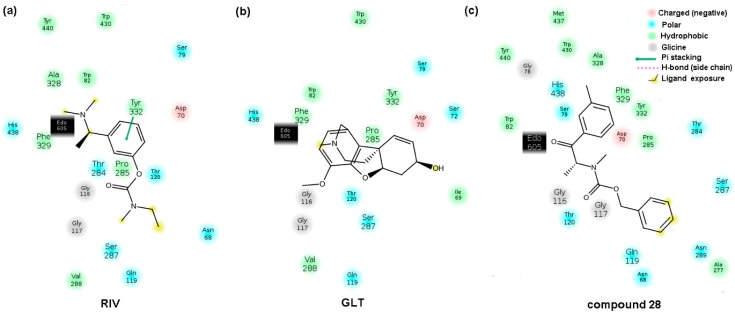
Planar (2D) comparision of the binding mode for: rivastigmine (**a**); galanthamine (**b**); and the most active BChE inhibitor **28** that was tested (**c**), as generated by the Maestro software.

**Figure 9:**
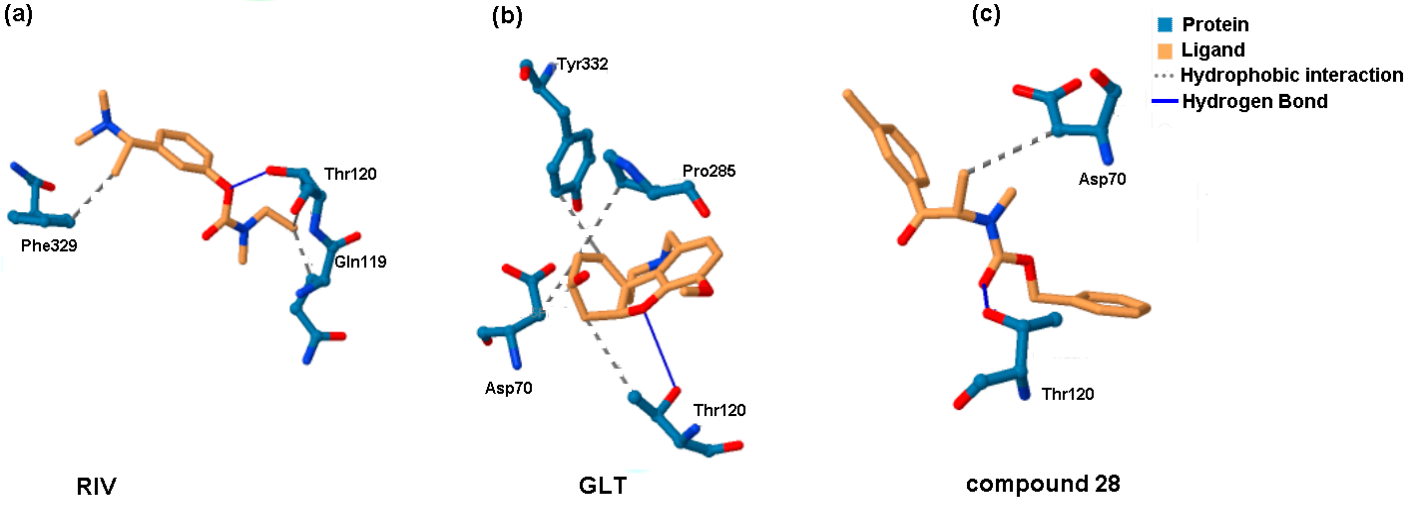
Spatial (3D) comparison of the binding mode for: rivastigmine (**a**); galanthamine (**b**); and the the most active BChE inhibitor **28** that was tested (**c**), as generated by the PLIP software.

Moreover, a run-through was performed to investigate the interacting mode for the most active compounds, which basically revealed two types of non-binding interactions: hydrophobic and hydrogen bond formation. Planar (2D) and spatial (3D) maps of the host–target interactions were created for all of the active compounds using Schrödinger Maestro software and the Protein-Ligand Interaction Profiler (PLIP) and were then compared with the drug (GLT and RIV) molecules [46]. Overall, the hydrophobic interactions were primarily generated with Asp70 (94%), Trp82 (28%), Pro285 (22%) and Tyr332 (22%), while Thr120 (83%) was basically specified as the hydrogen bond donor (HBD) that interacted with one of the oxygen atoms in the molecule carboxylic group. The 2D- and 3D-binding patterns for GLT, RIV and the most active BChE inhibitor (**28**) that was tested are illustrated in Figure 8 and Figure 9, respectively. The hydroxyl substituent of Thr120 appeared to be crucial to form the hydrogen bond with the ether (Figure 9a,b) or carbonyl oxygen (Figure 9c). For the examined BChE inhibitors, the regions close to the nitrogen atom (R^1^ substituent) seemed to be valid for the hydrophobic interactions with the Asp70 amino acid residue, which is in line with our previous receptor-independent findings (Figure 5).

Unfortunately, the obtained docking findings did not provide a clear explanation of the variations that the *meta/para*-positioned carbamate derivatives exerted on the enzyme reaction site; however, the close proximity of a positively charged nitrogen atom of His438 (Figure 8c) might have been potentially beneficial to the inhibition potential, especially the negatively charged chlorine- or bromine-based carbamates, which corresponds relatively well with the IVE-PLS CoMSA results. The electrostatic repulsion between the negatively charged atoms and the oxygen of Ser79 can partially explain the detrimental impact of the –OCH_3_ or –OCH_2_O- groups attached to the phenyl ring. On the other hand, the postulated hydrophobic interactions with Phe329 might favorably contribute to the inhibitory potential, as was observed for (di)methyl derivatives. 

Obviously, we should *beware of q^2^* [47] and *beware of docking* [48]; therefore, a combination of the consensus pharmacophore mapping with a systematic screening of multifaceted guest–host interactions with target-tailored procedures is highly advisable in rational drug design.

## 3. Materials and Methods

### 3.1. Chemistry

All of the reagents and solvents were purchased from ACROS Organics, Asta-Tech, Maybridge, Santa Cruz Biotechnology and Sigma-Aldrich. The melting points were measured in an OptiMelt—Automated Melting Point System (MPA 100, Stanford Research Systems). The ^1^H and ^13^C NMR spectra were recorded on a Bruker Ascend 500 MHz spectrometer at frequencies of 500 MHz and 126 MHz and a Bruker Avance III 400 MHz FT-NMR spectrometer at frequencies of 400 MHz and 101 MHz using CDCl_3_-d_6_ or DMSO-d_6_ as the solvent and TMS as the internal standard. The NMR solvents were purchased from ACROS Organics. The chemical shifts (δ) are given in ppm and the values of the coupling constants (J) are reported in hertz (Hz). The purity of all of the compounds was tested using the HPLC/MS method. The HPLC-MS analyses were performed on a Varian model 920 liquid chromatograph equipped with a Varian 900-LC model autosampler, a gradient pump, a Varian Pro Star 510 model column oven and a Varian 380-LC model evaporative light scattering detection (ELSD) detector. This was coupled with a Varian 500-MS IT. The HRMS were determined using a Waters LCT Premier XE high resolution mass spectrometer with electrospray ionization (ESI).

### 3.2. General Procedure Used to Synthesize Carbamates **1**–**41**

A mixture of a suitable acid chloride (1 moL) and an anhydrous aluminum chloride (1.05 moL) was stirred until the aluminum chloride was completely dissolved, and then cooled in a water bath and diluted in 250 mL of methylene chloride. Next, a suitable aromatic hydrocarbon (1.10 moL) was added dropwise to a magnetically stirred solution. The reaction mixture was poured into ice-cooled water, the dish was washed with distilled water three times and the organic layer was separated. Methylene chloride was evaporated and the reaction product was crystallized or purified by distillation. In the next step, 1.1 moL of bromine was added dropwise to the solution of the suitable aromatic ketone (1 moL) dissolved in 300 mL of methylene chloride. After the bromine was dropped into the magnetically stirred mixture, 300 mL of distilled water was added and mixed until the color of the mixture changed to white or cream. Then, the organic layer was separated and used in that form in the next step. A bromine derivative (1 moL) was dissolved in methylene chloride, a suitable amine (2 moL) was added to the obtained aromatic ketone and then sodium hydroxide was added dropwise. After 12 h of stirring, the organic layer was separated and poured into a beaker filled with ice and hydrochloric acid. An aqueous sodium hydroxide solution was then added to the separated aqueous phase. The extracted amine was washed three times with water, dried with anhydrous magnesium sulfate, diluted with diethyl ether and treated with gaseous hydrogen chloride until an acidic pH solution was obtained. The isolated hydrochloride of the 2-amino-1-phenylpropane-1-one derivative in the form of a white solid was washed with acetone and dried. Then, 0.01 mol of 2-amino-1-phenylpropane-1-one derivative dissolved in 50 mL of methylene chloride and 0.01 mol of triethyloamine was added to the cooled and magnetically stirred mixture, after which approximately 9 mmoL of suitable chloroformate was added dropwise. After dropping, the mixture was washed with diluted hydrochloric acid to remove any excess unreacted 1-phenylpropylamine derivative. The organic layer was separated and dried with anhydrous magnesium sulfate. The solvent was evaporated under reduced pressure and the product was purified on a column chromatography using hexane: ethyl acetate 9:1 as the eluent.

*Methyl ethyl(1-oxo-1-phenylpropan-2-yl)carbamate* (**1**). Yield 88%;^1^H-NMR (CDCl_3_, 400 MHz, ppm): δ 7.99 (t, H, aromat, J = 20.6 Hz); 7.83 (d, H, aromat, J_1_ = 51.2 Hz); 7.57 (ddd, H, aromat, J_1_ = 8.5 Hz, J_2_ = 2.4 Hz, J_3_ = 1.2 Hz); 7.46 (dd, H, aromat, J_1_ = 10.5 Hz, J_2_ = 4.7 Hz); 7.28 (s, H, aromat); 5.75 (d, H, CH, J = 6.6 Hz); 3.75 (s, 3H, CH_3_); 3.30 (s, H, CH_2_); 3.16 (m, H, CH_2_); 1.71–1.24 (m, 3H, CH_3_); 1.10–0.78 (m, 3H, CH_3_); ^13^C-NMR (CDCl_3_, 100 MHz, ppm): δ 199.2; 155.6; 135.6; 133.3; 128.6; 128.6; 128.4; 128.4; 55.2; 52.8; 38.5; 14.9; 14.3; HR-MS (ESI): for C_13_H_17_NO_3_Na [M+Na]^+^ calculated: 258.1106. m/z, found: 258.1102 m/z.

*Methyl methyl(1-oxo-1-phenylbutan-2-yl)carbamate* (**2**). Yield 83%; ^1^H-NMR (DMSO, 400 MHz, ppm): δ 7.90 (dd, 2H, aromat, J_1_ = 21.8 Hz, J_2_ = 7.5); 7.71–7.41 (m, 3H, aromat), 5.35 (ddd, 1H, CH, J_1_ = 52.6 Hz, J_2_ = 9.6 Hz, J_3_ = 5.1 Hz), 3.47 (d, 3H, CH_3_, J = 111.4 Hz), 2.79–2.44 (m, 2H, CH_2_), 1.92-1.62 (m, 3H, CH_3_), 1.05-0.72 (m, 3H, CH_3_); ^13^C-NMR (DMSO, 100 MHz, ppm): δ 204.0; 161.8; 140.7; 138.4; 133.9; 133.9; 133.1; 133.1; 65.9; 57.8; 34.9; 25.6; 15.5; HR-MS (ESI): for C_13_H_17_NO_3_Na [M+Na]^+^ calculated: 258.1106 m/z, found: 258.1103 m/z.

*Methyl ethyl(1-oxo-1-phenylbutan-2-yl)carbamate* (**3**). Yield 85%; ^1^H-NMR (CDCl_3_, 400 MHz, ppm): δ 8.01 (dd, 2H, aromat, J_1_ = 48.8 Hz, J_2_ = 6.8); 7.62–7.25 (m, 3H, aromat), 5.54 (dd, 1H, CH, J_1_ = 65.9 Hz, J_2_ = 58.5 Hz), 3.79 (d, 3H, CH_3_, J = 17.3 Hz), 3.14 (dd, 2H, CH_2_, J_1_ = 22,1 Hz, J_2_ = 7,1 Hz), 2.10–1.92 (m, 2H, CH_2_), 1.08–0.81 (m, 3H, CH_3_), 1.08-0.81 (m, 3H, CH_3_); ^13^C-NMR (CDCl_3_, 100 MHz, ppm): δ 198.8; 157.3; 135.9; 133.3; 128.5; 128.5; 127.9; 127.9; 60.1; 52.9; 38.1; 21.8; 15.0; 10.4; HR-MS (ESI): for C_14_H_19_NO_3_ Na [M+Na]^+^ calculated: 272.1263 m/z, found: 272.1263 m/z.

*Methyl methyl(1-oxo-1-(m-tolyl)propan-2-yl)carbamate* (**4**). Yield 86%; ^1^H-NMR (CDCl_3_, 400 MHz, ppm): 7.75 (dd, 2H, aromat, J_1_ = 42.0 Hz, J_2_ = 8,6); 7.44–7.28 (m, 2H, aromat); 5.79–5.25 (m, 1H, CH), 3.95–3.45 (m, 3H, CH_3_), 2.77 (d, 3H, CH_3_, J = 34,6 Hz), 2.30 (d, 3H, CH_3_, J = 91,9); 1.69–1.22 (m, 3H, CH_3_); ^13^C-NMR (CDCl_3_, 101 MHz, ppm): 199.5; 156.9; 138.5; 135.4; 134.1; 128.8; 128.5; 125.6; 55.0; 52.9; 29.3; 21.3; 13.5; HR-MS (ESI): for C_13_H_17_NO_3_ Na [M+Na]^+^ calculated: 258.1106 m/z, found: 258.1105 m/z.

*Methyl (1-(3-chlorophenyl)-1-oxopropan-2-yl)(methyl)carbamate* (**5**). Yield 89%; ^1^H-NMR (CDCl_3_, 500 MHz, ppm): 8.15–7.69 (m, 2H, aromat); 7.64–6.99 (m, 2H, aromat); 5.77–5.28 (m, 1H, CH), 3.76 (d, 3H, CH_3_ J = 16.0 Hz), 2.76 (d, 3H, CH_3_, J = 42.1 Hz), 1.46–1.19 (m, 3H, CH_3_); ^13^C-NMR (CDCl_3_, 126 MHz, ppm): 198.1; 156.8; 136.9; 135.0; 133.2; 130.0; 128.4; 126.5; 55.3; 53.1; 29.4; 13.3; HR-MS (ESI): for C_12_H_14_ClNO_3_ [M+H]^+^ calculated: 256.0740 m/z, found: 256.0741 m/z.

*Methyl (1-(4-methoxyphenyl)-1-oxopropan-2-yl)(methyl)carbamate* (**6**). Yield 79%; ^1^H-NMR (CDCl_3_, 400 MHz, ppm): 7.97 (dd, 2H, aromat, J_1_ = 42.0 Hz, J_2_ = 8.4); 7.01–6.87 (m, 2H, aromat); 5.84–5.14 (m, 1H, CH), 3.88 (s, 3H, CH_3_); 3.76 (d, 3H, CH_3_, J = 11.1 Hz), 2.93-2.58 (m, 3H, CH_3_), 1.37 (d, 3H, CH_3_, J = 6.9 Hz); ^13^C-NMR (CDCl_3_, 101 MHz, ppm): 197.3; 163.7; 156.8; 130.8; 130.5; 128.2; 113.9; 113.9; 55.4; 54.4; 53.0; 29.1; 13.4; HR-MS (ESI): for C_13_H_17_NO_4_ Na [M+Na]^+^ calculated: 274.1055 m/z, found: 274.1043 m/z.

*Methyl methyl(1-oxo-1-(p-tolyl)propan-2-yl)carbamate* (**7**). Yield 87%; m.p.: 36-37.5 °C; ^1^H-NMR (CDCl_3_, 500 MHz, ppm): 7.87 (dd, 2H, aromat, J_1_ = 53.2 Hz, J_2_ = 7.8); 7.28 (dd, 2H, aromat, J_1_ = 5.2 Hz, J_2_ = 4.6); 5.65 (dt, 1H, CH, J_1_ = 156.3 Hz, J_2_ = 6.8), 3.76 (d, 3H, CH_3_ J = 8.6 Hz), 2.76 (d, 3H, CH_3_, J = 49.2 Hz), 2.44 (d, 3H, CH_3_, J = 15.6); 1.81–0.70 (m, 3H, CH_3_); ^13^C-NMR (CDCl_3_, 126 MHz, ppm): 198.7; 156.8; 144.2; 132.8; 129.4; 129.4; 128.6; 128.6; 54.8; 53.0; 29.2; 21.7; 13.4; HR-MS (ESI): for C_13_H_17_NO_3_ Na [M+Na]^+^calculated: 258.1106, m/z, found: 258.1108 m/z.

*Methyl (1-(4-chlorophenyl)-1-oxopropan-2-yl)(methyl)carbamate* (**8**). Yield 87%; ^1^H-NMR (CDCl_3_, 500 MHz, ppm): 7.91 (dd, 2H, aromat, J_1_ = 55.8 Hz, J_2_ = 8.1); 7.50–7.16 (m, 2H, aromat); 5.72–5.31 (m, 1H, CH), 3.75 (s, 3H, CH_3_), 2.75 (d, 3H, CH_3_, J = 53.5 Hz), 1.49–1.23 (m, 3H, CH_3_); ^13^C-NMR (CDCl_3_, 126 MHz, ppm): 197.9; 156.8; 139.8; 133.6; 129.9; 129.9; 129.0; 129.0; 54.0; 53.1; 29.2; 13.2; HR-MS (ESI): for C_12_H_14_ClNO_3_ [M+H]^+^ calculated: 256.0740 m/z, found: 256.0743 m/z.

*Methyl (1-(4-bromophenyl)-1-oxopropan-2-yl)(methyl)carbamate* (**9**). Yield 87%; ^1^H-NMR (DMSO, 400 MHz, ppm): 7.77 (dd, 4H, aromat, J_1_ = 23.0 Hz, J_2_ = 8.6); 5,35 (dt, 1H, CH, J_1_ = 21.8 Hz, J_2_ = 6.7 Hz), 3.58–3.26 (m, 3H, CH_3_), 2.85–2.44 (m, 3H, CH_3_), 1.35–1.24 (m, 3H, CH_3_); ^13^C-NMR (DMSO, 100 MHz, ppm): 203.3; 160.8; 139.8; 136.9; 136.9 135.1; 135.1; 132.3; 61.9; 57.9; 35.8; 18.2; HR-MS (ESI): for C_12_H_14_BrNO_3_ [M+H]^+^ calculated: 300.0235 m/z, found: 300.0222 m/z.

*Methyl (1-(benzo[d][1,3]dioxol-5-yl)-1-oxopropan-2-yl)(methyl)carbamate* (**10**). Yield 76%; ^1^H-NMR (CDCl_3_, 500 MHz, ppm): 7.54 (ddd, 1H, aromat, J_1_ = 57.3 Hz, J_2_ = 45.8, J_3_ = 16.5); 7.28 (s, 1H, aromat); 6.87 (d, 1H, aromat, J = 8.2); 6.06 (s, 2H, CH_2_); 5.77–5.26 (m, 1H, CH), 3.77 (d, 3H, CH_3_, J = 15.2); 2.94–2.48 (m, 3H, CH_3_); 1.61 (s, 3H, CH_3_); ^13^C-NMR (CDCl_3_, 126 MHz, ppm): 197.0; 156.8; 152.4 148.2; 129.9; 125.0; 108.2; 101.8; 100,4; 54.5; 53.0; 29.1; 13.5; HR-MS (ESI): for C_13_H_15_NO_5_Na [M+Na]^+^ calculated: 288.0848 m/z, found: 288.0842 m/z.

*Phenyl ethyl(1-oxo-1-phenylpropan-2-yl)carbamate* (**11**). Yield 87%; ^1^H-NMR (CDCl_3_, 400 MHz, ppm): 8.03 (dd, 2H, aromat, J_1_ = 27.1 Hz, J_2_ = 7.5); 7.68–6.71 (m, 8H, aromat); 5.69 (dq, 1H, CH, J_1_ = 92.3, J_2_ = 6,8); 3.56-3.15 (m, 2H, CH_2_), 1.56 (dd, 3H, CH_3_, J_1_ = 36.3 Hz, J_2_ = 6.6); 1.20 (dt, 3H, CH_3_, J_1_ = 22.3 Hz, J_2_ = 7.1); ^13^C-NMR (CDCl_3_, 101 MHz, ppm): 199.1; 154.9; 151.4 135.5; 133.4; 129.4; 129.4; 128.8; 128.8; 128.5; 128.5; 125.5; 121.6; 121.6; 55.5; 39.1; 15.6; 14.6; HR-MS (ESI): for C_18_H_19_NO_3_ Na [M+Na]^+^ calculated: 320.1263m/z, found: 320.1258 m/z. 

*Phenyl methyl(1-oxo-1-phenylbutan-2-yl)carbamate* (**12**). Yield 89%; ^1^H-NMR (CDCl_3_, 500 MHz, ppm): 8.07 (ddd, 2H, aromat, J_1_ = 21.3 Hz, J_2_ = 7.4, J_3_ = 1.2); 7.65–7.59 (m, 1H, aromat); 7.51 (td, 2H, aromat, J_1_ = 7.4 Hz, J_2_ = 3.7); 7.43-7.35 (m, 2H, aromat); 7.29–7.20 (m, 1H, aromat); 7.14–7.09 (m, 2H, aromat); 5.62 (ddd, 1H, CH, J_1_ = 34.9, J_2_ = 9.5, J_3_ = 5.6); 2.91 (d, 3H, CH_3_, J = 32.3); 2.09–1.81 (m, 2H, CH_2_); 1.08 (dt, 3H, CH_3_, J_1_ = 35.9 Hz, J_2_ = 7.4); ^13^C-NMR (CDCl_3_, 126 MHz, ppm): 197.8; 155.5; 151.3 135.7; 133.5; 129.3; 129.3; 128.8; 128.8; 128.6; 128.6; 125.5; 121.7; 121.7; 60.4; 29.8; 20.9; 10.4; HR-MS (ESI): for C_18_H_19_NO_3_Na [M+Na]^+^ calculated: 320.1263 m/z, found: 320.1260 m/z.

*Phenyl ethyl(1-oxo-1-phenylbutan-2-yl)carbamate* (**13**). Yield 88%; ^1^H-NMR (CDCl_3_, 500 MHz, ppm): 8.21–7.90 (m, 2H, aromat); 7.62 (dd, 1H, aromat, J_1_ = 14.2 Hz, J_2_ = 6.9); 7.54–7.48 (m, 2H, aromat); 7.46–7.37 (m, 2H, aromat); 7.33–7.21 (m, 1H, aromat); 7.18–7.09 (m, 2H, aromat); 5.61 (ddd, 1H, CH, J_1_ = 44.6, J_2_ = 8.7, J_3_ = 6.1); 3.63–3.12 (m, 2H, CH_2_); 2.12-1.82 (m, 2H, CH_2_); 1.13 (td, 3H, CH_3_, J_1_ = 7.2 Hz, J_2_ = 3.4); 1.06 (dt, 3H, CH_3_, J_1_ = 14.8 Hz, J_2_ = 7.2); ^13^C-NMR (CDCl_3_, 126 MHz, ppm): 198.6; 155.4; 151.4 135.8; 133.5; 129.4; 129.4; 128.8; 128.8; 128.6; 128.6; 125.4; 121.6; 121.6; 60.4; 38.8; 21.8; 15.3; 10.5; HR-MS (ESI): for C_19_H_21_NO_3_Na [M+Na]^+^ calculated: 334.1419 m/z, found: 334.1411 m/z.

*Phenyl methyl(1-oxo-1-phenylpentan-2-yl)carbamate* (**14**). Yield 76%; ^1^H-NMR (CDCl_3_, 500 MHz, ppm): 8.11–8.02 (m, 2H, aromat); 7.62 (dd, 1H, aromat, J_1_ = 14.4 Hz, J_2_ = 7.2 Hz); 7.54–7.47 (m, 2H, aromat); 7.47–7.35 (m, 2H, aromat); 7.32–7.20 (m, 1H, aromat); 7.13–7.08 (m, 2H, aromat); 5.73 (ddd, 1H, CH, J_1_ = 28.4, J_2_ = 9.0, J_3_ = 5.8); 2.92 (d, 3H, CH_3_, J = 30.6); 1.98–1.81 (m, 2H, CH_2_); 1.49–1.40 (m, 2H, CH_2_); 1.04 (t, 3H, CH_3_, J = 7.4 Hz); ^13^C-NMR (CDCl_3_, 126 MHz, ppm): 199.0; 155.4; 151.4; 135.7; 133.5; 129.5; 129.5; 128.8; 128.8; 128.6; 128.6; 125.4; 121.7; 121.7; 58.6; 30.2; 29.8; 19.2; 13.9; HR-MS (ESI): C_19_H_21_NO_3_ [M+H]^+^ calculated for: 312.1600 m/z, found: 312.1605 m/z.

*Phenyl methyl(1-oxo-1-(m-tolyl)propan-2-yl)carbamate* (**15**). Yield 84%; m.p.: 52.5-53.5 °C; ^1^H-NMR (CDCl_3_, 400 MHz, ppm): 7.94–7.73 (m, 2H, aromat); 7.40 (dt, 2H, aromat, J_1_ = 15.7 Hz, J_2_ = 7.5); 7.28 (s, 2H, aromat); 7.23 (t, 1H, aromat, J = 7.4 Hz); 7.09 (t, 2H, aromat, J = 8.3 Hz); 5.74 (dq, 1H, CH, J_1_ = 50.8, J_2_ = 6.9); 2.94 (d, 3H, CH_3_, J = 10.2 Hz); 2.44 (s, 3H, CH_3_); 1.48 (d, 3H, CH_3_, J = 7,0 Hz); ^13^C-NMR (CDCl_3_, 101 MHz, ppm): 199.3; 154.9; 151.4; 138.6; 135.4; 134.3; 129.3; 129.3 128.9; 128.7; 125.7; 125.4; 121.6; 121.6; 55.4; 30.0; 21.3; 13.6; HR-MS (ESI): for C_18_H_19_NO_3_Na [M+Na]^+^ calculated: 320.1263 m/z, found: 320.1277 m/z.

*Phenyl (1-(3-chlorophenyl)-1-oxopropan-2-yl)(methyl)carbamate* (**16**). Yield 92%; ^1^H-NMR (CDCl_3_, 500 MHz, ppm): 8.01 (dd, 1H, aromat, J_1_ = 7.5 Hz, J_2_ = 5.7 Hz); 7.91 (dd, 1H, aromat, J_1_ = 27.4Hz, J_2_ = 7.8 Hz); 7.63–7.56 (m, 1H, aromat); 7.49–7.42 (m, 1H, aromat); 7.42–7.35 (m, 2H, aromat); 7.27–7.19 (m, 1H, aromat); 7.13–7.06 (m, 2H, aromat); 5.68 (dq, 1H, CH, J_1_ = 61.2, J_2_ = 6.9); 2.93 (d, 3H, CH_3_, J = 17.1 Hz); 1.49 (d, 3H, CH_3_, J = 6.9 Hz); ^13^C-NMR (CDCl_3_, 126 MHz, ppm): 198.0; 155.0; 151.3; 137.0; 135.1; 133.5; 130.2; 129.4; 129.4; 128.5; 126.5; 125.6; 121.6; 121.6; 55.6; 30.0; 13.4; HR-MS (ESI): for C_17_H_16_ClNO_3_ Na [M+Na]^+^ calculated: 340.0717 m/z, found: 340.0714 m/z.

*Phenyl (1-(4-methoxyphenyl)-1-oxopropan-2-yl)(methyl)carbamate* (**17**). Yield 78%; m.p.: 53–56 °C; ^1^H-NMR (CDCl_3_, 400 MHz, ppm): 8.04 (dd, 2H, aromat, J_1_ = 17.9 Hz, J_2_ = 8.8); 7.39 (dd, 2H, aromat, J_1_ = 10.7 Hz, J_2_ = 5.1); 7.30–7.19 (m, 1H, aromat); 7.11 (t, 2H, aromat, J = 8.8 Hz); 6.97 (d, 2H, aromat, J = 8.9 Hz); 5.74 (dq, 1H, CH, J_1_ = 53.9, J_2_ = 6.9); 3.90 (s, 3H, CH_3_); 2.93 (d, 3H, CH_3_, J = 8,2 Hz); 1.47 (d, 3H, CH_3_, J = 6.7 Hz); ^13^C-NMR (CDCl_3_, 101 MHz, ppm): 199.3; 154.9; 151.4; 138.6; 135.4; 134.3; 129.3; 129.3 128.9; 128.7; 125.7; 125.4; 121.6; 121.6; 55.4; 30.0; 21.3; 13.6; HR-MS (ESI): for C_18_H_19_NO_4_Na [M+Na]^+^ calculated: 336.1212 m/z, found: 336.1222 m/z.

*Phenyl methyl(1-oxo-1-(p-tolyl)propan-2-yl)carbamate* (**18**). Yield 82%; m.p.: 42.5–44 °C; ^1^H-NMR (CDCl_3_, 500 MHz, ppm): 7.94 (dd, 2H, aromat, J_1_ = 22.3 Hz, J_2_ = 8.2); 7.39 (t, 2H, aromat, J = 7.9 Hz); 7.30 (dd, 2H, aromat, J_1_ = 11.7 Hz, J_2_ = 3.7); 7.23 (t, 1H, aromat, J = 7.4 Hz); 7.14–7.07 (m, 2H, aromat); 5.74 (dq, 1H, CH, J_1_ = 72.3, J_2_ = 6.9); 2.93 (d, 3H, CH_3_, J = 9.3 Hz); 2.44 (s, 3H, CH_3_); 1.48 (d, 3H, CH_3_, J = 6.9 Hz); ^13^C-NMR (CDCl_3_, 126 MHz, ppm): 198.5; 154.9; 151.4; 144.4; 132.8; 129.5; 129.5; 129.3; 129.3; 128.7; 128.7; 125.5; 121.7; 121.7; 55.0; 29.9; 21.7; 13.6; HR-MS (ESI): for C_18_H_19_NO_3_Na [M+Na]^+^ calculated: 320.1263 m/z, found: 320.1262 m/z.

*Phenyl (1-(4-chlorophenyl)-1-oxopropan-2-yl)(methyl)carbamate* (**19**). Yield 91%; m.p.: 52–54 °C; ^1^H-NMR (CDCl_3_, 500 MHz, ppm): 7.98 (dd, 2H, aromat, J_1_ = 27.4 Hz, J_2_ = 8.5 Hz); 7.48 (dd, 2H, aromat, J_1_ = 11.8 Hz, J_2_ = 4.9Hz); 7.43–7.36 (m, 2H, aromat); 7.24 (t, 1H, aromat, J = 7.4 Hz); 7.08 (dd, 2H, aromat, J_1_ = 22.8 Hz, J_2_ = 7.8 Hz); 5.68 (dq, 1H, CH, J_1_ = 82.3, J_2_ = 6.9); 2.93 (s, 3H, CH_3_); 1.48 (d, 3H, CH_3_, J = 6.9 Hz); ^13^C-NMR (CDCl_3_, 126 MHz, ppm): 197.7; 154.9; 151.3; 140.0; 133.7; 130.0; 130.0; 129.4; 129.4; 129.1; 129.1; 125.6; 121.6; 121.6; 55.2; 29.9; 13.4; HR-MS (ESI): for C_17_H_16_ClNO_3_Na [M+Na]^+^ calculated: 340.0717 m/z, found: 340.0712 m/z.

*Phenyl (1-(4-bromophenyl)-1-oxopropan-2-yl)(methyl)carbamate* (**20**). Yield 88%; m.p.: 54–55 °C; ^1^H-NMR (CDCl_3_, 400 MHz, ppm): 7.90 (dd, 2H, aromat, J_1_ = 21.9 Hz, J_2_ = 8.5 Hz); 7.65 (dd, 2H, aromat, J_1_ = 8.3 Hz, J_2_ = 6.5 Hz); 7.40 (dd, 2H, aromat, J_1_ = 11.0 Hz, J_2_ = 4.8 Hz); 7.25 (dd, 1H, aromat, J_1_ = 15.9 Hz, J_2_ = 8.5 Hz); 7.08 (dd, 2H, aromat, J_1_ = 18.7 Hz, J_2_ = 7.8 Hz); 5.67 (dq, 1H, CH, J_1_ = 66.0, J_2_ = 6.9); 2.93 (s, 3H, CH_3_); 1.48 (d, 3H, CH_3_, J = 6.9 Hz); ^13^C-NMR (CDCl_3_, 101 MHz, ppm): 198.0; 155.0; 151.3; 134.1; 132.1; 132.1; 130.0; 130.0; 129.4; 129.4; 128.8; 125.6; 121.6; 121.6; 55.2; 29.9; 13.4; HR-MS (ESI): for C_17_H_16_BrNO_3_Na [M+Na]^+^ calculated: 384.0211 m/z, found: 384.0226 m/z.

*Phenyl (1-(3,4-dimethylphenyl)-1-oxopropan-2-yl)(methyl)carbamate* (**21**). Yield 74%; m.p.: 94.5–95.5 °C; ^1^H-NMR (CDCl_3_, 500 MHz, ppm): 7.84–7.74 (m, 2H, aromat); 7.42–7.36 (m, 2H, aromat); 7.29–7.19 (m, 2H, aromat); 7.11 (dd, 2H, aromat, J_1_ = 11.6, J_2_ = 4.1); 5.75 (dq, 1H, CH, J_1_ = 57.6, J_2_ = 6.9); 2.92 (d, 3H, CH_3_, J = 14.5); 2.41–2.25 (m, 6H, CH_3_); 1.47 (d, 3H, CH_3_, J = 6.9); ^13^C-NMR (CDCl_3_, 126 MHz, ppm): 198.8; 154.9; 151.4; 143.2; 137.2; 133.2; 130.0; 129.6; 129.3; 129.3; 126.3; 125.4; 121.7; 121.7; 55.1; 29.9; 20.1; 19.8; 13.7; HR-MS (ESI): for C_19_H_21_NO_3_Na [M+Na]^+^ calculated: 334.1419 m/z, found: 334.1418 m/z.

*Phenyl (1-(benzo[d][1,3]dioxol-5-yl)-1-oxopropan-2-yl)(methyl)carbamate* (**22**). Yield 76%; m.p.: 94–95 °C; ^1^H-NMR (CDCl_3_, 500 MHz, ppm): 7.75–7.63 (m, 1H, aromat); 7.54–7.48 (m, 1H, aromat); 7.39 (dd, 2H, aromat, J_1_ = 10.8 Hz, J_2_ = 5.1); 7.24 (dt, 1H, aromat, J_1_ = 13.8 Hz, J_2_ = 5.9Hz); 7.12 (dd, 2H, aromat, J_1_ = 12.5 Hz, J_2_ = 4.8 Hz); 6.89 (dd, 1H, aromat, J_1_ = 8.2 Hz, J_2_ = 4.9 Hz); 6.08 (d, 2H, CH_2_, J = 7.7 Hz); 5.69 (dq, 1H, CH, J_1_ = 58.8, J_2_ = 6.9); 2.93 (d, 3H, CH_3_, J = 19.9 Hz); 1.46 (d, 3H, CH_3_, J = 6.9 Hz); ^13^C-NMR (CDCl_3_, 126 MHz, ppm): 196.8; 154.9; 152.2; 151.4; 148.4; 129.1; 129.4; 129.4; 125.5; 125.0; 121.7; 121.7; 108.2; 108,0; 102.0; 54.7; 29.8; 13.7; HR-MS (ESI): for C_18_H_17_NO_5_Na [M+Na]^+^ calculated: 350.1004 m/z, found: 350.0998 m/z.

*Benzyl ethyl(1-oxo-1-phenylpropan-2-yl)carbamate* (**23**). Yield 82%; ^1^H-NMR (CDCl_3_, 500 MHz, ppm): 8.01 (d, 2H, aromat, J = 7.6); 7.81 (d, 1H, aromat, J = 7.0 Hz); 7.60–7.26 (m, 7H, aromat); 5.76 (q, 1H, CH, J = 6.8 Hz); 5.31–5.05 (m, 2H, CH_2_); 3.32–3.10 (m, 2H, CH_2_), 1.43 (t, 3H, CH_3_, J = 8.0); 1.05–0.98 (m, 3H, CH_3_); ^13^C-NMR (CDCl_3_, 101 MHz, ppm): 199.3; 156.2; 136.7 135.5; 133.3; 128.7; 128.7; 128.5; 128.5; 128.5; 128.5; 128.0; 127.6; 127.6; 67.4; 55.4; 38.7; 15.5; 14.6; HR-MS (ESI): for C_19_H_21_NO_3_ [M+H]^+^ calculated: 312.1600 m/z, found: 312.1645 m/z.

*Benzyl methyl(1-oxo-1-phenylbutan-2-yl)carbamate* (**24**). Yield 87%; ^1^H-NMR (d_6_-aceton, 500 MHz, ppm): 8.03-7.87 (m, 2H, aromat); 7.62 (dt, 1H, aromat, J_1_ = 22.0, J_2_ = 7.4); 7.52–7.29 (m, 7H, aromat); 5.52 (ddd, 1H, CH, J_1_ = 46.7, J_2_ = 9.6, J_3_ = 5.3); 5.29–5.10 (m, 2H, CH_2_); 2.76 (s, 3H, CH_3_); 1.99–1.73 (m, 2H, CH_2_); 0.98-0.92 (m, 3H, CH_3_); ^13^C-NMR (d_6_-aceton, 126 MHz, ppm): 198.4; 156.4; 137.3 136.1; 133.1; 128.6; 128.6; 128.4; 128.4; 128.2; 128.2; 127.8; 127.5; 127.5; 66.8; 60.7; 29.6; 20.5; 9.8; HR-MS (ESI): for C_19_H_21_NO_3_Na [M+Na]^+^ calculated: 334.1419 m/z, found: 334.1418 m/z.

*Benzyl ethyl(1-oxo-1-phenylbutan-2-yl)carbamate* (**25**). Yield 83%; ^1^H-NMR (CDCl_3_, 500 MHz, ppm): 8.10-7.83 (m, 2H, aromat); 7.60-7.26 (m, 8H, aromat); 5.62 (dd, 1H, CH, J_1_ = 8.3, J_2_ = 6.6); 5.22 (q, 2H, CH_2_, J = 12.5 Hz) 3.14 (dd, 2H, CH_2_, J_1_ = 14.1, J_2_ = 7.0); 2.07–1.72 (m, 2H, CH_2_); 0.99 (dq, 6H, 2CH_3_, J_1_ = 13.5 Hz, J_2_ = 6.9); ^13^C-NMR (CDCl_3_, 126 MHz, ppm): 198.8; 156.7; 136.7; 135.9; 133.3; 128.7; 128.7; 128.5; 128.5; 128.5; 128.5; 128.0; 127.6; 127.6; 67.5; 60.3; 38.3; 21.7; 15.1; 10.5; HR-MS (ESI): for C_20_H_23_NO_3_ [M+H]^+^ calculated: 326.1756 m/z, found: 326.1763 m/z.

*Benzyl methyl(1-oxo-1-phenylpentan-2-yl)carbamate* (**26**). Yield 80%; ^1^H-NMR (CDCl_3_, 500 MHz, ppm): 8.06-7.82 (m, 2H, aromat); 7.56 (dt, 1H, aromat, J_1_ = 23.9 Hz, J_2_ = 7.4 Hz); 7.48–7.27 (m, 7H, aromat); 5.60 (ddd, 1H, CH, J_1_ = 115.5, J_2_ = 9.2, J_3_ = 5.6); 5.33-5.12 (m, 2H, CH_2_); 2.78 (d, 3H, CH_3_, J = 15.1); 1.93-1.72 (m, 2H, CH_2_); 1.43-1.30 (m, 2H, CH_2_); 0.99 (dt, 3H, CH_3_, J_1_ = 15.0 Hz, J_2_ = 7.4 Hz); ^13^C-NMR (CDCl_3_, 126 MHz, ppm): 199.0; 156.7; 136.7; 135.7; 133.3; 128.7; 128.7; 128.5; 128.5; 128.3; 128.3; 128.0; 127.6; 127.6; 67.5; 58.5; 29.7; 29.3; 19.1; 13.9; HR-MS (ESI): for C_20_H_23_NO_3_Na [M+Na]^+^ calculated: 348.1576 m/z, found: 348.1574 m/z.

*Benzyl methyl(1-oxo-1-(m-tolyl)propan-2-yl)carbamate* (**27**). Yield 81%; ^1^H-NMR (CDCl_3_, 400 MHz, ppm): 7.83–7.58 (m, 2H, aromat); 7.41–7.18 (m, 7H, aromat); 5.63 (dq, 1H, CH, J_1_ = 104.2, J_2_ = 6.8); 5.29-5.10 (m, 2H, CH_2_); 2.80 (d, 3H, CH_3_, J = 22.4 Hz); 2.36 (d, 3H, CH_3_, J = 25.2 Hz); 1.41 (d, 3H, CH_3_, J = 6.9); ^13^C-NMR (CDCl_3_, 101 MHz, ppm): 199.4; 156.2; 138.5; 136.7; 135.4; 134.1; 128.9; 128.5; 128.5; 128.5; 128.5; 128.0; 127.6; 125.6; 67.4; 55.2; 29.4; 21.3; 13.5; HR-MS (ESI): for C_19_H_21_NO_3_Na [M+Na]^+^ calculated: 334.1419 m/z, found: 334.1419 m/z.

*Benzyl (1-(3-chlorophenyl)-1-oxopropan-2-yl)(methyl)carbamate* (**28**). Yield 91%; ^1^H-NMR (CDCl_3_, 500 MHz, ppm): 8.00–7.86 (m, 1H, aromat); 7.84–7.63 (m, 1H, aromat); 7.56–7.49 (m, 1H, aromat); 7.47–7.44 (m, 1H, aromat); 7.42–7.23 (m, 5H, aromat); 5.55 (dq, 1H, CH, J_1_ = 141.1, J_2_ = 6,8); 5.27–5.11 (m, 2H, CH_2_); 2.79 (d, 3H, CH_3_, J = 30.2 Hz); 1.41 (d, 3H, CH_3_, J = 6.9 Hz); ^13^C-NMR (CDCl_3_, 126 MHz, ppm): 198.0; 156.2; 136.9; 136.5; 136.1; 135.0; 133.2; 130.0; 128.4; 128.4; 128.7; 127.7; 127.7; 126.5; 67.6; 55.5; 29.5; 13.3; HR-MS (ESI): for C_18_H_18_ClNO_3_Na [M+Na]^+^ calculated: 354.0873 m/z, found: 354.0863 m/z.

*Benzyl (1-(4-methoxyphenyl)-1-oxopropan-2-yl)(methyl)carbamate* (**29**). Yield 79%; m.p.: 48–50 °C; ^1^H-NMR (CDCl_3_, 400 MHz, ppm): 7.91 (dd, 2H, aromat, J_1_ = 74.8 Hz, J_2_ = 8.6); 7.43–7.24 (m, 5H, aromat); 6.85 (dd, 2H, aromat, J_1_ = 55.0 Hz, J_2_ = 8.6); 5.67 (dt, 1H, CH, J_1_ = 93.9, J_2_ = 6.8); 5.36-5.08 (m, 2H, CH_2_); 3.87 (d, 3H, CH_3_, J = 9.7 Hz); 2.77 (d, 3H, CH_3_, J = 14.7 Hz); 1.42-1.35 (m, 3H, CH_3_); ^13^C-NMR (CDCl_3_, 101 MHz, ppm): 197.3; 163.7; 156.2; 136.7; 130.9; 130.9; 128.5; 128.5; 128.2; 128.0 127.7; 127.7; 113.9; 113.9; 67.4; 55.4; 54.6; 29.2; 13.4; HR-MS (ESI): for C_19_H_21_NO_4_ [M+H]^+^ calculated: 328.1549 m/z, found: 328.1537 m/z.

*Benzyl methyl(1-oxo-1-(p-tolyl)propan-2-yl)carbamate* (**30**). Yield 84%; m.p.: 62–63 °C; ^1^H-NMR (d_6_-aceton, 500 MHz, ppm): 7.89-7.74 (m, 2H, aromat); 7.42-7.21 (m, 7H, aromat); 5.60 (dq, 1H, CH, J_1_ = 44.4, J_2_ = 6.9); 5.24-5.04 (m, 2H, CH_2_); 2.86–2.76 (m, 3H, CH_3_); 2.07 (tt, 3H, CH_3_, J_1_ = 4.4, J_2_ = 2.2); 1.43-1.33 (m, 3H, CH_3_); ^13^C-NMR (d_6_-aceton, 126 MHz, ppm): 198.1; 155.7; 143.8; 137.3; 133.2; 129.2; 129.2; 128.4; 128.4; 128.3; 128.3; 127.8; 127.5; 127.5; 66.8; 55.6; 30.0; 20.7; 12.8; HR-MS (ESI): for C_19_H_21_NO_3_Na [M+Na]^+^ calculated: 334.1419 m/z, found: 334.1419 m/z.

*Benzyl (1-(4-chlroophenyl)-1-oxopropan-2-yl)(methyl)carbamate* (**31**). Yield 90%; m.p.: 72–73 °C; ^1^H-NMR (d_6_-aceton, 500 MHz, ppm): 7.90 (dd, 2H, aromat, J_1_ = 50.6 Hz, J_2_ = 8.4 Hz); 7.47 (dd, 2H, aromat, J_1_ = 38.9 Hz, J_2_ = 48.4 Hz); 7.40–7.27 (m, 5H, aromat); 5.53 (dq, 1H, CH, J_1_ = 26.0, J_2_ = 6.7); 5.22–5.02 (m, 2H, CH_2_); 2.87–2.76 (m, 3H, CH_3_); 1.39 (t, 3H, CH_3_, J = 8.3 Hz); ^13^C-NMR (d_6_-aceton, 126 MHz, ppm): 197.6; 155.7; 138.5; 137.2; 134.6; 129.9; 129.9; 128.7; 128.7; 128.4 128.4; 127.9; 127.9; 127.5; 66.8; 56.3; 29.7; 12.5; HR MS (ESI): for C_18_H_18_ ClNO_3_Na [M+Na]^+^ calculated: 354.0873 m/z, found: 354.0864 m/z.

*Benzyl (1-(4-bromophenyl)-1-oxopropan-2-yl)(methyl)carbamate* (**32**). Yield 92%; m.p.: 63–64 °C; ^1^H-NMR (d_6_-aceton, 500 MHz, ppm): 7.90–7.74 (m, 2H, aromat); 7.63 (dd, 2H, aromat, J_1_ = 38.6 Hz, J_2_ = 8.3 Hz); 7.40–7.27 (m, 5H, aromat); 5.57–5.45 (m, 1H, CH); 5.22–5.02 (m, 2H, CH_2_); 2.82 (dd, 3H, CH_3_, J_1_ = 12.5 Hz, J_2_ = 4.2 Hz); 1.39 (t, 3H, CH_3_, J = 8.2 Hz); ^13^C-NMR (d_6_-aceton, 126 MHz, ppm): 197.8; 155.7; 137.2; 134.9; 131.7; 131.7; 129.9; 129.9; 128.4; 128.4; 127.8; 127.5; 127.5; 127.2; 66.8; 56.3; 29.8; 12.5; HR-MS (ESI): for C_18_H_18_BrNO_3_ Na [M+Na]^+^ calculated: 398.0368 m/z, found: 398.0367 m/z.

*Benzyl (1-(3,4-dimethylphenyl)-1-oxopropan-2-yl)(methyl)carbamate* (**33**). Yield 79%, ^1^H-NMR (d_6_-aceton, 500 MHz, ppm): 7.78–7.59 (m, 2H, aromat); 7.41–7.28 (m, 5H, aromat); 7.21 (dd, 1H, aromat, J_1_ = 30.0 Hz, J_2_ = 7.8 Hz); 5.61 (dq, 1H, CH, J_1_ = 45.9, J_2_ = 6.8); 5.15 (ddd, 2H, CH_2_, J_1_ = 31.2 Hz, J_2_ = 15.7 Hz, J_3_ = 8.9 Hz) 2.88-2.74 (m, 3H, CH_3_); 2.29 (dd, 3H, CH_3_, J_1_ = 24.4 Hz, J_2_ = 15.7 Hz); 2.07 (ddt, 3H, CH_3_, J_1_ = 6.6 Hz, J_2_ = 4.4 Hz, J_3_ = 2.2 Hz) 1.41-1.33 (m, 3H, CH_3_); ^13^C-NMR (d_6_-aceton, 126 MHz, ppm): 198.2; 155.7; 142.5; 137.3; 136.9; 133.6; 129.7; 129.7; 129.3; 128.4; 128.4; 127.8; 127.5; 125.9; 66.7; 55.5; 30.0; 19.1; 18.9; 12.9; HR-MS (ESI): for C_20_H_23_NO_3_Na [M+Na]^+^ calculated: 348.1576 m/z, found: 348.1575 m/z.

*Benzyl (1-(benzo[d][1,3]dioxol-5-yl)-1-oxopropan-2-yl)(methyl)carbamate* (**34**). Yield 79%; m.p.: 60–61.5 °C; ^1^H-NMR (CDCl_3_, 500 MHz, ppm): 7.67 (dd, 1H, aromat, J_1_ = 8.2 Hz, J_2_ = 1.4 Hz); 7.51-7.26 (m, 6H, aromat); 6.85-6.64 (m, 1H, aromat); 6.06 (s, 2H, CH_2_); 5.58 (dq, 1H, CH, J_1_ = 130.5, J_2_ = 6.8); 5.33-5.12 (m, 2H, CH_2_) 2.78 (d, 3H, CH_3_, J = 20.4 Hz); 1.44-1.33 (m, 3H, CH_3_); ^13^C-NMR (CDCl_3_, 126 MHz, ppm): 197.0; 156.2; 152.0; 148.2; 136.6; 130.0; 128.5; 128.5; 128.0; 127.7; 127.7; 125.0; 108.3; 108.1; 101.8; 67.5; 54.6; 29.2; 13.6; HR MS (ESI): for C_19_H_19_NO_5_ [M+H]^+^ calculated: 342.1342 m/z, found: 342.1354 m/z.

*Methyl methyl(1-(naphthalen-2-yl)-1-oxopropan-2-yl)carbamate* (**35**). Yield 69%; ^1^H-NMR (CDCl_3_, 400 MHz, ppm): 8.41 (dd, 1H, aromat, J_1_ = 29.5, Hz, J_2_ = 8.3); 8.00 (d, 2H, aromat, J = 8.3 Hz); 7.93–7.87 (m, 1H, aromat); 7.63–7.46 (m, 3H, aromat); 5.80-5.29 (m, 1H, CH), 3.68 (d, 3H, CH_3_, J = 13.5); 2.87 (d, 3H, CH_3_, J = 42.2 Hz); 1.53-1.42 (m, 3H, CH_3_); ^13^C-NMR (CDCl_3_, 101 MHz, ppm): 203.7; 157.1; 134.2 134.0; 132.5; 130.5; 128.5; 127.8; 127.2; 126.4; 125.3; 124.5; 58.0; 52.9; 30.0; 13.6; HR-MS (ESI): for C_16_H_17_NO_3_Na [M+Na]^+^ calculated: 294.1106 m/z, found: 294.1107 m/z.

*Phenyl (1naphthalen-1-yl)-1-oxopropan-2-yl)(methyl)carbamate* (**36**). Yield 68%; ^1^H-NMR (CDCl_3_, 400 MHz, ppm): 8.38 (dd, 1H, aromat, J_1_ = 56.8, Hz, J_2_ = 8.6); 7.94 (ddd, 3H, aromat, J_1_ = 28.4 Hz, J_2_ = 14.9 Hz, J_3_ = 7.9 Hz); 7.65-7.44 (m, 3H, aromat); 7.41-7.22 (m, 5H, aromat); 5.63 (gq, 1H, CH; J_1_ = 103.9, Hz, J_2_ = 7.0); 2.95–2.82 (m, 3H, CH_3_); 1.50–1.45 (m, 3H, CH_3_); ^13^C-NMR (CDCl_3_, 101 MHz, ppm): 203.5; 156.4; 136.6; 134.2; 134.0; 132.5; 130.6; 128.5; 128.5; 128.5; 128.5; 127.9; 127.8; 127.6; 127.2; 126.4; 125.3; 124.5; 58.2; 30.2; 13.6; HR-MS (ESI): for C_21_H_19_NO_3_ [M+H]^+^ calculated: 334.1443 m/z, found: 334.1450 m/z.

*Phenyl (1-(4-chloronaphthalen-1-yl)-1-oxopropan-2-yl)(methyl)carbamate* (**37**). Yield 71%; m.p.: 85-87 °C; ^1^H-NMR (CDCl_3_, 500 MHz, ppm): 8.50-8.35 (m, 2H, aromat); 7.87 (dd, 1H, aromat, J_1_ = 73.5 Hz, J_2_ = 7.8 Hz); 7.72–7.58 (m, 3H, aromat); 7.39–7.18 (m, 3H, aromat); 7.06–6.92 (m, 2H, aromat) 5.66 (dq, 1H, CH, J_1_ = 63.0 Hz, J_2_ = 7.0 Hz); 3.03 (d, 3H, CH_3_, J = 15.8); 1.54 (d, 3H, CH_3_, J = 7.1 Hz); ^13^C-NMR (CDCl_3_, 126 MHz, ppm): 202.8; 155.2; 151.3; 136.7; 133.4; 131.7; 131.3; 129.3; 129.3; 128.6; 127.6; 127.0; 125.7; 125.5; 125.0; 124.8; 121.6; 121.6; 58.6; 30.9; 13.6; HR-MS (ESI): for C_21_H_18_ClNO_3_ [M+H]^+^ calculated: 368.1053 m/z, found: 368.1067 m/z.

*Phenyl (1-(4-bromonaphthalen-1-yl)-1-oxopropan-2-yl)(methyl)carbamate* (**38**). Yield 72%; m.p.: 103–104 °C; ^1^H-NMR (CDCl_3_, 500 MHz, ppm): 8.46–8.30 (m, 2H, aromat); 7.88-7.83 (m, 1H, aromat); 7.72–7.56 (m, 3H, aromat); 7.39–7.19 (m, 3H, aromat); 7.06–6.91 (m, 2H, aromat); 5.64 (dq, 1H, CH, J_1_ = 61.2 Hz, J_2_ = 7.1 Hz); 3.03 (d, 3H, CH_3_, J = 16.6); 1.54 (d, 3H, CH_3_, J = 7.1 Hz); ^13^C-NMR (CDCl_3_, 126 MHz, ppm): 203.0; 155.2; 151.2; 134.3; 132.5; 131.6; 129.3; 129.3; 128.8; 128.6; 127.9; 127.8; 127.1; 126.2; 125.7; 125.5; 121.6; 121.6; 58.7; 30.9; 13.6; HR MS (ESI): for C_21_H_18_BrNO_3_Na [M+Na]^+^ calculated: 434.0368 m/z, found: 434.0352 m/z.

*Benzyl methyl(1-(naphthalen-1-yl)-1-oxopropan-2-yl)carbamate* (**39**). Yield 68%; ^1^H-NMR (CDCl_3_, 500 MHz, ppm): 8.48–8.27 (m, 1H, aromat); 7,.(ddd, 3H, aromat; J_1_ = 35.0 Hz, J_2_ = 16.7 Hz, J_3_ = 9.3); 7.65–7.45 (m, 3H, aromat); 7.41–7.22 (m, 5H, aromat); 5.63 (dq, 1H, CH, J_1_ = 130.5 Hz, J_2_ = 7.0 Hz); 5.24–5.02 (m, 2H, CH_2_); 2.89 (d, 3H, CH_3_, J = 33.7 Hz); 1.48 (dd, 3H, CH_3_, J_1_ = 7.0 Hz, J_2_ = 3.9 Hz); ^13^C-NMR (CDCl_3_, 126 MHz, ppm): 203.6; 156.4; 136.6; 134.2; 134.0; 132.6; 130.5; 128.5; 128.5; 127.9; 127.8; 127.8; 127.6; 127.6; 127.2; 126.4; 125.3; 124.5; 67.4; 58.2; 30.2; 13.6; HR-MS (ESI): for C_22_H_21_NO_3_ [M+H]^+^ calculated: 348.1600 m/z, found: 348.1608 m/z.

*Benzyl (1-(4-chloronaphthalen-1-yl)-1-oxopropan-2-yl)(methyl)carbamate* (**40**). Yield 72%; m.p.: 65–66 °C; ^1^H-NMR (CDCl_3_, 400 MHz, ppm): 8.50–8.28 (m, 2H, aromat); 7.87 (d, 1H, aromat; J = 7.8 Hz,); 7.70–7.46 (m, 3H, aromat); 7.43-7.19 (m, 5H, aromat); 5.55 (dq, 1H, CH, J_1_ = 91.4 Hz, J_2_ = 7.0 Hz); 5.26-4.98 (m, 2H, CH_2_); 2.88 (d, 3H, CH_3_, J = 30.1 Hz); 1.48 (t, 3H, CH_3_, J = 5.8 Hz); ^13^C-NMR (CDCl_3_, 101 MHz, ppm): 202.9; 156.3; 136.5; 136.4; 133.3; 131.7; 131.3; 128.7; 128.5; 128.5; 128.5; 128.5; 128.0; 127.6; 126.9; 126.2; 125.8; 124.9; 67.4; 58.3; 30.3; 13.4; HR-MS (ESI): for C_22_H_20_ClNO_3_Na [M+Na]^+^ calculated: 404.1029 m/z, found: 404.1014 m/z.

*Benzyl (1-(4-bromonaphthalen-1-yl)-1-oxopropan-2-yl)(methyl)carbamate* (**41**). Yield 73%; m.p.: 59–60 °C; ^1^H-NMR (CDCl_3_, 400 MHz, ppm): 8.47–8.22 (m, 2H, aromat); 7.78 (d, 1H, aromat; J = 7.9 Hz,); 7.70–7.51 (m, 3H, aromat); 7.44–7.16 (m, 5H, aromat); 5.54 (dq, 1H, CH, J_1_ = 89.1 Hz, J_2_ = 7,0 Hz); 5.26-4.96 (m, 2H, CH_2_); 2.88 (d, 3H, CH_3_, J = 29.8 Hz); 1.48 (t, 3H, CH_3_, J = 6.1 Hz); ^13^C-NMR (CDCl_3_, 101 MHz, ppm): 203.0; 156.3; 136.5; 134.1; 132.5; 131.6; 128.7; 128.7; 128.5; 128.5; 128.5; 128.5; 128.0; 127.8; 127.7; 127.6; 127.0; 125.8; 67.4; 58.4; 30.4; 13.4; HR-MS (ESI): for C_22_H_20_BrNO_3_ Na [M+Na]^+^ calculated: 448.0524 m/z, found: 448.0512 m/z.

### 3.3. Evaluating the In Vitro AChE- and BChE-Inhibition Potency 

The ability of all the prepared compounds to inhibit AChE from electric eel (*Electrophorus electricus*) and BChE from equine serum (both purchased from Sigma, St. Louis, MO, USA) was determined in vitro using a modified Ellman’s method. The effectiveness of the inhibitors, which are expressed as the IC_50_ values, represent the concentration of an inhibitor that was required for reduction of enzyme activity (or reaction rate) to 50% (sometimes referred to as the negative logarithm of the molar concentration required to inhibit the enzyme activity by 50%, pIC_50_ = log 1/IC_50_). The Ellman’s method is widely used for measuring cholinesterase activity and the effectivity of ChEIs [49]. It is a simple, quick and direct method to determine the content of the SH and –S–S– groups in proteins [50]. Cholinesterase activity is measured indirectly by quantifying the concentration of the 5-thio-2-nitrobenzoic acid (TNB) ion that is formed in the reaction between disulfide reagent 5,5′-dithiobis-2-nitrobenzoic acid (DTNB) and thiocholine, which is a product of substrate (i.e., acetylthiocholine, ATCh) hydrolysis that is catalyzed by cholinesterase [51].

All of the examined compounds were dissolved in DMSO (concentration 0.01 M) and diluted in demineralized water (concentrations 0.001 M and 0.0001 M). The ability of the tested compounds to inhibit AChE (from electric eel) and BChE (from equine serum) was determined using a modified Ellman’s method at 25 °C in the presence of phosphate buffered saline (PBS, 0.1 M, pH 7.4) in a glass cuvette with a 1 cm optical path. The enzyme activity in the total reaction mixture (2 mL) was 0.2 U/mL, the concentration of the substrate ATCh 40 μM and the concentration of DTNB 0.1 mM for all of the reactions. The IC_50_ values were obtained from the dependence of *v*_0_/*v*_i_ on the concentration of the tested compound (inhibitor), where *v*_0_ is the reaction rate of an uninhibited reaction and *v_i_* is the reaction rate of an inhibited reaction (for a given concentration of the inhibitor). First, *v*_0_ was determined. PBS (0.1 M, pH 7.4), DTNB and ATCh were put into the cuvette. The enzymatic reaction was started by adding the enzyme. The dependence of absorbance (λ = 412 nm) on time was observed for 70 s (the reference solution contained PBS, DTNB and ATCh), and then the reaction rate (*v*_0_) was calculated (*v* = Δ*A*/Δ*t*). The measurement was performed at least in triplicate, and average *v*_0_ was determined. Then, *v*_i_ (for a given concentration of the inhibitor) was determined. DTNB, ATCh and the selected volume of a suitably diluted inhibitor (to achieve the required concentration of the inhibitor in the total reaction mixture) and a certain volume of PBS (to achieve the total volume of the reaction mixture 2 mL after adding the enzyme) were put into the cuvette. The enzymatic reaction was started by adding the enzyme. The dependence of absorbance (λ = 412 nm) on time was observed for 70 s (the reference solution was the same as for uninhibited reaction), and then the reaction rate (*v*_i_) was calculated. To determine the IC_50_ values, twelve different concentrations of the inhibitor were used and each measurement was performed at least in triplicate. Finally, the dependence of *v*_0_/*v*_i_ on the concentration of the inhibitor was determined, and the IC_50_ values were calculated from the obtained equation of the regression curve for y = 2 (resulting from the definition of the IC_50_ value) [52]. The obtained results are presented in Table 1.

### 3.4. Comparative Molecular Surface Analysis using Iterative PLS-Based Variable Elimination

Self-organizing neural mapping (SOM) is regarded as being a nonlinear projection tool that decreases the dimensionality of the input object, e.g., converts 3D objects to 2D, while preserving the topological relationships between the input and output data. Moreover, a trained network can be used to project the specified molecular property (expressed as a vector) by generating a 2D color-coded clustering pattern that is called a feature map. Hence, the SOM algorithm was used to generate an electrostatic potential map in the form of a 2D topographic pattern produced from input signals (points) that were sampled randomly at the molecular surface [53]. In this case, specifying the closest neighbor and then projecting the signals into this particular neuron is based on a comparison of each 3D input vector that consists of *x*, *y* and *z* coordinates with a three-element weight vector that describes each neuron. The shape of a specific molecular surface (template) that is encoded in the weights of the trained Kohonen network can be used to process the signals coming from the surface of the other molecule(s) (counter-template), thereby producing a series of comparative SOM maps can be used to compare/contrast the superimposed molecular geometry. 

Applying SOMs to compress/visualize/classify the structural data has been widely reported, e.g., for the 2D mapping of the electrostatic potential on 3D molecular surfaces or partial atomic charges for atomic molecular representation [54].

The set of CoMSA shape/electronic descriptors is subsequently processed using the PLS method and expresses the relationship between variable *y* and the set of predictors *X* in a form that is represented by the following equation:

*y* = *X* × *b* + e (1)

where *b* is the vector of the regression coefficients and *e* is the vector of the errors. Generally, PLS models are constructed for centered/autoscaled data, and their complexity is estimated using, e.g., the leave-one-out cross-validation (LOO-CV) procedure. The cross-validated $q_{cv}^{2}$ is calculated as:

(2) $$q_{cv}^{2}=1-\frac{\sum_{i}^{m}{\left(obs_{i}-pred_{i}\right)}^{2}}{\sum_{i}^{m}{\left(obs_{i}-mean\left(obs_{i}\right)\right)}^{2}}$$

where *obs* is the observed value; *pred* is the predicted value; *mean* is the mean value of *obs*; and *i* refers to the object index, which ranges from 1 to *m*.

The quality of the external predictions was measured using the standard deviation of error of the prediction (SDEP), $q_{test}^{2}$ and the mean absolute error (MAE) parameters, which are defined, respectively, as:

(3) $$SDEP=\sqrt{\frac{\sum_{i}^{n}{\left(pred_{i}-obs_{i}\right)}^{2}}{n}}$$

(4) $$q_{cv}^{2}=1-\frac{\sum_{i}^{n}{\left(obs_{i}-pred_{i}\right)}^{2}}{\sum_{i}^{n}{\left(obs_{i}-mean\left(obs_{i}\right)\right)}^{2}}$$

(5) $$MAE=\frac{\sum_{i}^{n}\left|pred_{i}-obs_{i}\right|}{n}$$

where *n* is the number of objects in a test set.

Redundant variables can impede the interpretation of a model by increasing its complexity; therefore, reducing the number of variables is advisable. The iterative variable elimination (IVE-PLS) procedure, which is a modification of the UVE-PLS algorithm (developed by Centner et al.) has been proposed, to analyze the stability of the regression coefficients, which are expressed as the *mean(b)/s(b)* ratio, where *s(b)* represents the standard deviation of the regression coefficient *b* [55]. Generally, the entire procedure is composed of the following steps: (i) a standard PLS analysis with LOO-CV to assess the performance of the PLS model ($q_{cv}^{2}$); (ii) elimination of the matrix column with the lowest stability value; (iii) a standard PLS analysis of the new matrix without the column that was eliminated in Step (ii); and (iv) the recurrent repetition of Steps (i)–(iii) to maximize the LOO $q_{cv}^{2}$ parameter.

A molecule might be encoded by an ensemble of structural (S) and physicochemical (P) properties that are organized in a vector, which represents an object in the chemical space (CS). The distribution of the empirically (FCS) and virtually (VCS) produced compounds can be visually scrutinized using, e.g., a linear projection procedure called Principal Component Analysis (PCA). PCA is regarded as being a classical method to explore data that permits the data dimensionality to be reduced, visualized and the relationships between the objects (molecules) and parameters (descriptors) to be interpreted. On the condition that the reduction of the data dimensionality is efficient, it is possible to capture interesting information about the data structure (uncover groups of objects and atypical objects) to indicate the importance of the original data variables that contribute to the observed structure, and finally, to illustrate and interpret the relationships between the objects and the parameters in the X matrix (using the first few principal components PCs) [56,57].

### 3.5. Building the Model and Molecular Modeling

The same laboratory was employed to specify all of the pharmacological data to eliminate any potential data noise that might have been introduced by pooling the datasets from various sources. The in vitro AChE and BChE inhibition values (IC_50_) for the set of carbamate derivatives are listed in Table 1. The CACTVS/csed molecular editor was used to build the set of the respective compound models. The spatial geometry of the molecules was specified using a CORINA 3D generator. The (inter)change file format converter OpenBabel was used to convert the chemical data.

The majority of the modeling studies were performed with a Sybyl-X 2.0/Certara software package running on a HP workstation with a Debian 6.0 operating system. The standard Tripos force field (POWELL conjugate gradient algorithm) with a 0.01 kcal/mol energy gradient convergence criterion and a distant dependent dielectric constant was used to optimize the initial geometry of each compound (MAXMIN2 module). For the electrostatic potential calculations, the Gasteiger–Hückel method (implemented in Sybyl-X) was initially used to produce the partial atomic charges. One 13-ordered atom trial alignment on **32** was selected to cover the entire bonding topology in the maximal common structure (MCS) using the atom FIT method, which is based on matching the positions of the atoms between the corresponding atom pairs.

The SONNIA software was used in the CoMSA analysis to simulate 20 × 20 to 50 × 50 SOMs with a winning distance that varied within a range of 0.2–2.0. The Cartesian coordinates of the molecular surfaces for the superimposed molecules were produced by a SOM network to form a 2D map of the electrostatic potential. Pretty active against AChE and BChE **32** was selected to form the template molecules. The output maps were subsequently transformed into a 400–2500-element vector, which was used by the PLS method implemented in the MATLAB programming environment. 

The crystallographic structure of BChE, which contained one amino acid chain and rivastigmine analog molecule, was retrieved from the PDB repository (code 6eul). Only the 1,2-ethanediol molecule was retained, because it was specified as being valid in the enzyme active site AC2. The rest of the heteroatoms, including the crystallographic waters, were extracted prior to the calculations. The ligand/protein structures for the docking study were prepared in the pdbqt file format with the Gasteiger charges calculated. During the AutoDock simulation, various poses (default nine) were generated progressively from a single conformer (an energy-optimized molecule) by applying a collection of the preferred torsion angles to the rotatable bonds and were evaluated using the united-atom scoring function. All of the predicted binding 2D/3D modes, including the positions of the flexible side chains, were visualized using PyMol, Maestro and VMD molecular graphics viewers and the Protein-Ligand Interaction Profiler (PLIP).

## 4. Conclusions

To summarize, a series of novel benzene-based derivatives was designed, synthesized and characterized using ^1^H NMR spectroscopy and HRMS. All 41 of the tested compounds were evaluated for their in vitro ability to potentially inhibit AChE and BChE, respectively. The selectivity index of the individual molecules to cholinesterases was also specified. Roughly speaking, a rather moderate inhibitory effect against AChE was revealed; however, some of the compounds (**11**, **13**, **14**, **16**, **23**–**28**, **31**, and **33**) proved to be very selective for the BChE enzyme. In fact, two compounds (**23** and **28**) had a very high selectivity index (SI = 13.93 and 15.31). Specifically, compound **28** had the lowest IC_50_ value revealing an approximately seven-fold higher inhibitory activity against BChE than RIV (IC_50_ = 5.51 vs. 38.40 μM), which corresponds quite well with GLT (IC_50_ = 2.77 μM). It was observed that the methyl-substituted carbamates generally exhibited a lower inhibitor ability compared to their phenyl or benzyl counterparts. Interestingly, compounds **17** and **29,** which had a methoxy group in the same position, were weaker inhibitors—the presence of the hydrophilic electron-donating -OCH_3_ substituent of the phenyl ring at the *para*-position decreased the potency of the compounds, especially against the BChE enzyme. On the other hand, the presence of methyl group(s) in the *meta*/*para*-position(s) of the phenyl ring (compounds **15**, **18**, **21**, **27**, **30**, and **33**) resulted in an improvement of the IC_50_ value for the BChE enzyme, thus suggesting the significance of a hydrophobic character for the interactions with the enzyme. Placing the electron-withdrawing chlorine substituent in the *meta*-position of the phenyl ring appeared to be strongly preferable, especially for the BChE inhibition activity (compounds **16** or **28**). To partially explain the observed variations in the *anti*-AChE/BChE potential of the investigated carbamate series, comparative receptor-independent (RI) and receptor-dependent (RD) structure–activity studies were conducted, respectively. The principal purpose of the ligand-based study was to comparatively analyze the molecular surface (CoMSA) to gain insight into the electronic and/or steric factors that govern the ability of the tested compounds to inhibit the AChE/BChE activities. The findings for the surface descriptors were compared with their force field counterparts (CoMFA) when modeling the inhibiting potency for multiple training/test subsets using the stochastic model validation (SMV) procedure. Moreover, a similarity analysis was performed using the PCA approach on the pool of Dragon descriptors. The spatial distribution of the potentially important steric and electrostatic factors on the BChE inhibitory potency was specified using the probability-guided pharmacophore mapping procedure based on the iterative variable elimination (IVE-PLS) method. Finally, a comprehensive scrutiny of the guest–target interactions for the inhibitors that were comparatively active to rivastigmine BChE (IC_50_ < 30 μM) was conducted using the site-directed computer-assisted docking methodology. Planar (2D) and spatial (3D) maps of the host–target interactions were created for all of the active compounds and compared with the marketed drug (GLT and RIV) molecules, which generally revealed two types of non-binding interactions—hydrophobic and hydrogen bond formation, respectively. The hydroxyl substituent of Thr120 appeared to be crucial in forming the hydrogen bond with the ether or carbonyl oxygen (Figure 9c). For the examined BChE inhibitors, the regions in close proximity to the nitrogen atom (R^1^ substituent) seemed to be valid for hydrophobic interactions with the Asp70 amino acid residue, which is in line with ligand-based findings. Regrettably, a clear explanation of the variations that the *meta/para*-positioned carbamate derivatives exerted on the BChE reaction site was not revealed; however, some regularities were observed in the ligand–receptor interaction pattern. It appeared that the close proximity of the positively charged nitrogen atom of His438 may potentially be beneficial to the inhibition potential, especially the negatively charged chlorine- or bromine-based carbamates, which corresponds relatively well with the IVE-PLS CoMSA results. The electrostatic repulsion between the negatively charged atoms and the oxygen of Ser79 can partially explain the detrimental impact of the –OCH_3_ or –OCH_2_O- groups that were attached to the phenyl ring. On the other hand, the postulated hydrophobic interactions with Phe329 can favorably contribute to the inhibitory potential, as was observed for (di)methyl derivatives. 

The combination of consensus pharmacophore mapping with a systematic screening of the multifaceted guest–host interactions using target-tailored approaches seems to be the path towards an intelligent drug design system.

## Figures and Tables

**Figure 7:**
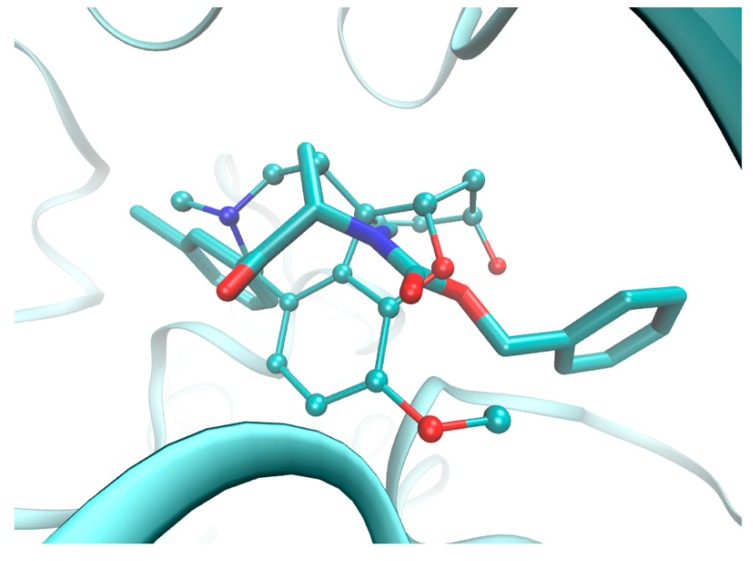
Structure of the BChE enzyme (displayed as ribbons) bound with GLT (depicted as balls and sticks), RIV (presented as thin sticks) and the most active molecule **28** (illustrated as thick sticks).

**Table 1:** Structures of the carbamate derivatives 1–41; in vitro AChE and BChE inhibition (IC_50_) compared with the standards rivastigmine (RIV) and galanthamine (GLT). ChE inhibitions are expressed as the mean ± SD (*n* = three experiments).

**Benzene derivatives** 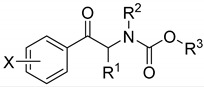							
**No.**	**X**	**R^1^**	**R^2^**	**R^3^**	**AChE** **IC_50_ [µM]**	**BChE** **IC_50_ [µM]**	SI *
**methyl carbamates**							
**1.**	H	CH_3_	C_2_H_5_	CH_3_	245.33 ± 0.75	227.91 ± 0.90	1.08
**2.**	H	C_2_H_5_	CH_3_	CH_3_	328.86 ± 9.72	440.56 ± 7.54	0.75
**3.**	H	C_2_H_5_	C_2_H_5_	CH_3_	273.74 ± 2.16	362.87 ± 7.41	0.75
**4.**	3-CH_3_	CH_3_	CH_3_	CH_3_	133.98 ± 2.30	115.89 ± 2.16	1.16
**5.**	3-Cl	CH_3_	CH_3_	CH_3_	329.68 ± 42.06	113.61 ± 0.29	2.90
**6.**	4-OCH_3_	CH_3_	CH_3_	CH_3_	146.91 ± 1.51	186.60 ± 1.55	0.79
**7.**	4-CH_3_	CH_3_	CH_3_	CH_3_	50.63 ± 0.63	180.77 ± 11.99	0.28
**8.**	4-Cl	CH_3_	CH_3_	CH_3_	308.80 ± 6.16	131.57 ± 4.55	2.35
**9.**	4-Br	CH_3_	CH_3_	CH_3_	282.74 ± 0.71	224.08 ± 2.19	1.26
**10.**	3,4-OCH_2_O	CH_3_	CH_3_	CH_3_	225.46 ± 2.72	194.93 ± 1.93	1.16
**phenyl carbamates**							
**11.**	H	CH_3_	C_2_H_5_	Ph	166.72 ± 1.75	27.64 ± 0.55	**6.03**
**12.**	H	C_2_H_5_	CH_3_	Ph	179.02 ± 6.21	52.96 ± 1.06	3.38
**13.**	H	C_2_H_5_	C_2_H_5_	Ph	95.69 ± 5.37	15.62 ± 0.06	**6.13**
**14.**	H	C_3_H_7_	CH_3_	Ph	97.97 ± 0.36	16.55 ± 0.13	**5.92**
**15.**	3-CH_3_	CH_3_	CH_3_	Ph	165.62 ± 2.75	48.29 ± 0.31	3.43
**16.**	3-Cl	CH_3_	CH_3_	Ph	108.81 ± 1.10	14.81 ± 0.01	**7.35**
**17.**	4-OCH_3_	CH_3_	CH_3_	Ph	90.21 ± 0.73	105.73 ± 1.94	0.85
**18.**	4-CH_3_	CH_3_	CH_3_	Ph	138.64 ± 1.45	87.63 ± 2.37	1.58
**19.**	4-Cl	CH_3_	CH_3_	Ph	44.24 ± 2.35	38.96 ± 0.28	1.14
**20.**	4-Br	CH_3_	CH_3_	Ph	51.46 ± 1.38	44.05 ± 1.71	1.17
**21.**	3,4-CH_3_	CH_3_	CH_3_	Ph	47.95 ± 2.09	26.41 ± 0.05	1.82
**22.**	3,4-OCH_2_O	CH_3_	CH_3_	Ph	84.00 ± 0.43	53.56 ± 0.72	1.57
**benzyl carbamates**							
**23.**	H	CH_3_	C_2_H_5_	Bn	158.41 ± 0.87	11.37 ± 0.05	**13.93**
**24.**	H	C_2_H_5_	CH_3_	Bn	104.15 ± 2.91	21.38 ± 0.26	**4.87**
**25.**	H	C_2_H_5_	C_2_H_5_	Bn	119.47 ± 1.23	21.29 ± 0.48	**5.61**
**26.**	H	C_3_H_7_	CH_3_	Bn	134.65 ± 0.70	16.30 ± 0.17	**8.26**
**27.**	3-CH_3_	CH_3_	CH_3_	Bn	83.32 ± 2.49	12.50 ± 0.24	**6.67**
**28.**	3-Cl	CH_3_	CH_3_	Bn	84.37 ± 2.27	5.51 ± 0.20	**15.31**
**29.**	4-OCH_3_	CH_3_	CH_3_	Bn	62.57 ± 0.65	57.39 ± 0.02	1.09
**30.**	4-CH_3_	CH_3_	CH_3_	Bn	40.06 ± 0.01	26.43 ± 0.40	1.52
**31.**	4-Cl	CH_3_	CH_3_	Bn	52.83 ± 0.34	7.02 ± 0.15	**7.53**
**32.**	4-Br	CH_3_	CH_3_	Bn	32.01 ± 0.54	11.28 ± 0.15	2.84
**33.**	3,4-CH_3_	CH_3_	CH_3_	Bn	76.78 ± 2.76	16.87 ± 0.22	**4.55**
**34.**	3,4-OCH_2_O	CH_3_	CH_3_	Bn	75.57 ± 1.25	51.89 ± 1.18	1.46
**Naphthalene derivatives** 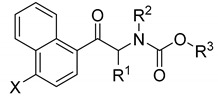							
**methyl carbamates**							
**35.**	H	CH_3_	CH_3_	CH_3_	126.82 ± 1.74	150.36 ± 7.13	0.84
**phenyl carbamates**							
**36.**	H	CH_3_	CH_3_	Ph	99.58 ± 5.88	12.72 ± 0.19	**7.83**
**37.**	Cl	CH_3_	CH_3_	Ph	30.64 ± 1.57	13.54 ± 0.47	2.26
**38.**	Br	CH_3_	CH_3_	Ph	61.68 ± 1.42	12.97 ± 0.28	**4.76**
**benzyl carbamates**							
**39.**	H	CH_3_	CH_3_	Bn	105.20 ± 0.99	18.94 ± 0.18	**5.55**
**40.**	Cl	CH_3_	CH_3_	Bn	103.28 ± 0.41	42.75 ± 0.31	2.42
**41.**	Br	CH_3_	CH_3_	Bn	128.27 ± 0.88	235.91 ± 16.3	0.54
**RIV**					56.1 ± 1.41	38.4 ± 1.97	1.46
**GLT**					1.54 ± 0.02	2.77 ± 0.15	0.56

* SI (index selectivity) = IC_50_ (AChE)/IC_50_ (BChE). A higher selectivity to BChE is indicated in bold.

## Abbreviations

AChE	Acetylcholinesterase
BChE	Butyrylcholinesterase
CAMD	Computer Asisted Molecular Design
ADMET	Absorption Distribution Metabolism Excretion Toxicity
CoMSA	Comparative Molecular Surface Analysis
SMV	Stochastic Model Validation
RIV	Rivastigmine
GLT	Galanthamine
PCA	Principal Component Analysis
IVE-PLS	Iterative Variable Elimination Partial Least Squares
PLIP	Protein Ligand Interaction Profiler
